# Inflammation and regulatory T cell genes are differentially expressed in peripheral blood mononuclear cells of Parkinson’s disease patients

**DOI:** 10.1038/s41598-021-81961-7

**Published:** 2021-01-27

**Authors:** Zerrin Karaaslan, Özlem Timirci Kahraman, Elif Şanlı, Hayriye Arzu Ergen, Canan Ulusoy, Başar Bilgiç, Vuslat Yılmaz, Erdem Tüzün, Haşmet Ayhan Hanağası, Cem İsmail Küçükali

**Affiliations:** 1 Department of Neuroscience, Aziz Sancar Institute of Experimental Medicine, Istanbul University, Istanbul, Turkey; 2 Department of Molecular Medicine, Aziz Sancar Institute of Experimental Medicine, Istanbul University, Istanbul, Turkey; 3 Department of Neurology, Istanbul Faculty of Medicine, Istanbul University, Istanbul, Turkey

**Keywords:** Neuroimmunology, Transcriptomics

## Abstract

Our aim was to identify the differentially expressed genes (DEGs) in peripheral blood mononuclear cells (PBMC) of Parkinson’s disease (PD) patients and healthy controls by microarray technology and analysis of related molecular pathways by functional annotation. Thirty PD patients and 30 controls were enrolled. Agilent Human 8X60 K Oligo Microarray was used for gene level expression identification. Gene ontology and pathway enrichment analyses were used for functional annotation of DEGs. Protein–protein interaction analyses were performed with STRING. Expression levels of randomly selected DEGs were quantified by real time quantitative polymerase chain reaction (RT-PCR) for validation. Flow cytometry was done to determine frequency of regulatory T cells (Tregs) in PBMC. A total of 361 DEGs (143 upregulated and 218 downregulated) were identified after GeneSpring analysis. DEGs were involved in 28 biological processes, 12 cellular components and 26 molecular functions. Pathway analyses demonstrated that upregulated genes mainly enriched in p53 (CASP3, TSC2, ATR, MDM4, CCNG1) and PI3K/Akt (IL2RA, IL4R, TSC2, VEGFA, PKN2, PIK3CA, ITGA4, BCL2L11) signaling pathways. TP53 and PIK3CA were identified as most significant hub proteins. Expression profiles obtained by RT-PCR were consistent with microarray findings. PD patients showed increased proportions of CD49d^+^ Tregs, which correlated with disability scores. Survival pathway genes were upregulated putatively to compensate neuronal degeneration. Bioinformatics analysis showed an association between survival and inflammation genes. Increased CD49d^+^ Treg ratios might signify the effort of the immune system to suppress ongoing neuroinflammation.

## Introduction

Parkinson’s disease (PD) is the second most common neurodegenerative disease after Alzheimer’s disease and affects 1% of the population above 60 year-old^1^. Accumulation of α-synuclein in intracellular deposits named Lewy bodies and loss of dopaminergic neurons in the substantia nigra pars compacta (SNpc) are the core pathological features of the disease^2^. Depletion of dopamine causes dysfunction of basal ganglia and leads to the classical motor symptoms of PD including bradykinesia, rigidity, resting tremor and postural instability. PD is also associated with numerous non-motor symptoms that might precede motor symptoms^3^. Although diagnosis of PD mainly relies on clinical findings, moderate to severe dopaminergic neuron loss occurs before the onset of motor symptoms^4^. Thus, it is crucial to diagnose the disease at early stages and develop disease-modifying treatments that will reduce neurodegeneration. Understanding of cellular mechanisms leading to loss of dopaminergic neurons might provide a basis for identification of diagnostic biomarkers and improvement of new therapeutic strategies for PD.

Etiopathogenesis of PD is not fully understood but environmental factors like pesticides, toxins, metals, head injury and certain drugs have received great attention for years^5^. However recent studies revealed that genetic susceptibility has a key role in PD pathogenesis and complex interactions of genetic and environmental factors are necessary for development of PD^6^. Monogenic forms of PD constitute < 10% of all cases but identification of these genes provides insight into molecular mechanisms underlying the disease^7^. Several studies established that oxidative stress, mitochondrial dysfunction and impairment of protein homeostasis contribute to disease mechanisms^8^. Neuroinflammation also seems to play a role in PD, but whether it induces harmful effects due to release of proinflammatory cytokines or has a protective role in terms of clearance of extracellular debris and production of trophic factors has not yet been established^9^. Evidence of CD4^+^ T cell infiltration in postmortem studies of PD brain specimens and increased expression of inflammatory cytokines have strengthened the idea that inflammation could be a prominent feature of PD^10,11^.

Microarray technology allows comparison of numerous gene expression profiles between healthy subjects and patients and thus provides a common strategy for analysis of neurodegenerative diseases. Several microarray studies have been carried out using both brain tissue and peripheral blood to date and gene expression profiles of PD have been established^12–17^. Blood-based gene expression analyses revealed altered expression of genes associated with ubiquitination/proteasomal system, mitochondrial function, oxidation and metabolism in PD patients as compared to controls^15^. Blood transcriptomics of drug-naïve sporadic PD showed increased expression of genes that are involved in leukocyte activation and epigenetic alterations that regulate chromatin remodelling^16^. Comparison of PD patients with LRRK2 mutation idiopathic PD cases and controls revealed altered expression of genes that are associated with Akt signaling pathway and B cell differentiation^18^. Tumor necrosis factor (TNF) signaling pathway was also found to be a key pathway involved in PD pathogenesis^19^. Analyses of differentially expressed gene profiles in experimental mouse model of MPTP induced PD revealed increased expression of systemic inflammation and programmed cell death genes^20^ Zhang et al. reported decreased expression of complex I transcripts and increased expression of heat shock proteins and some anti-apoptotic gene groups in PD patients^21^. Durrenberger et al. showed upregulation of P2X7 and NOS pathways thereby emphasizing that extracellular ATP and reactive astrocytes are responsible of microglial activation and subsequent release of proinflammatory cytokines that contribute to dopaminergic cell death^22^. In another microarray study, expression levels of neuro-immune signaling related transcripts were found to be increased in nucleated blood cells of PD patients^23^. Overall, these results indicate importance of neuroinflammation in PD pathogenesis.

To bring light to possible pathological mechanisms underlying PD and discover biomarkers associated with progression of motor symptoms in PD, we identified differentially expressed genes through microarray and transcriptome studies. Our results pinpointed expression alterations particularly in inflammation and survival genes. Notably, peripheral blood ratios of a subset of regulatory T cells (Tregs) showed association with altered disability scores of PD.

## Materials and methods

### Patient selection

Thirty consecutive idiopathic PD patients who were diagnosed by movement disorders specialists according to the United Kingdom Parkinson’s Disease Society Brain Bank Criteria and 30 controls with no prior history of neurological and inflammatory disease were enrolled (Table 2). All patients were under dopaminergic drug treatment. Exclusion criteria were presence of accompanying inflammatory or autoimmune diseases and being under immunosuppressive treatment. Included patients were evaluated by a neurologist, and each patient received the Unified Parkinson Disease Rating Scale (UPDRS) and the Hoehn and Yahr (H&Y) Scale.

### Microarray expression profiling and DEGs screening

Eighteen PD patients (12 males, mean age ± SD was 58.28 ± 9.45) and 18 control subjects (15 males, mean age ± SD was 48.54 ± 8.22) from the original group were enrolled for transcriptome studies. Mean years of disease duration of patients ± SD was 6.87 ± 4.08, mean total UPDRS scores ± SD was 34.94 ± 18.09, mean UPDRS III score ± SD was 17.50 ± 12.08 and mean H&Y score ± SD was 2.05 ± 0.64. Total RNA was extracted from whole blood samples by using Qiagen RNeasy (CatNo: 74104) kit according to the manufacturer’s protocol. Assessment of total RNA quality was performed by the Agilent 2100 Expert Software that automatically provides RNA Integrity Number (RIN). The minimum RIN number was defined as 7. Agilent Human 8 × 60 K Oligo Microarray has been used for gene level expression identification by manufacturer’s protocol (Agilent Technologies, Palo Alto, CA). Cyanine-3 was used to label targets, after the labeling, the samples were purified by using Absolutely RNA Nanoprep Kit (Agilent) and purified RNA samples were hybridized with Hybridization Kit (Agilent) at 65 °C for 17 h. After washing steps, array slide was put into the Agilent slide holder. After generating the microarray scan images, .tif images were extracted by using the Feature Extraction Software (FE) 4.0.1.21. After normalization by using Robust Multi-array Average (RMA) method, differentially expressed genes (DEGs) between PD and healthy controls were analyzed by Genespring. The significance level was set at p < 0.05 and log2 fold change > 2 for identifying DEGs.

### Gene ontology and pathway enrichment analyses

Gene ontology analysis (GO) provides functionally annotation of DEGs within a biological context with respect of biological processes, cellular components and molecular functions^24^. Kyoto Encyclopedia of Genes and Genomes (KEGG; http://www.kegg.jp/) pathway database was used for analyzing gene functions and to link this data with molecular pathways^25^. GO terms and KEGG pathway enrichment analyses for DEGs were performed by using the database for annotation, visualization and integrated discovery (DAVID) online database (DAVID Bioinformatics Resources 6.8).

### Protein–protein interaction

Search Tool for the Retrieval of Interacting Genes/Proteins (STRING; http://www.string-db.org/) is a database that identifies known and predicted interactions between proteins^26^. Selected pathways were filtered into the DEG protein–protein interaction (PPI) network complex containing nodes and edges with parameters including a minimum required interaction score > 0.4 (medium confidence). Only query proteins were displayed. Hub genes, highly interconnected with nodes in a module, have been considered functionally significant. A hub is a node that has a higher degree than other nodes in the graph. In our study, hub genes were defined by module connectivity. Furthermore, we uploaded all genes in the hub module to the STRING database to construct PPI to screen hub nodes in PPI network. We defined genes with the node connectivity > 2 (total edges/total nodes) as the hub nodes in PPI network. Additionally, the genes with p value less than 0.05 were identified as real hub genes.

### Validation of expression levels of DEGs

Expression levels of DEGs were confirmed by real time quantitative polymerase chain reaction (RT-PCR) studies for randomly selected 5 genes (PRKACB, TSC2, GATA2, PI3KCA, CASP3). Venous blood samples were collected in 10 ml EDTA containing tubes that were obtained from 8 randomly selected PD patients (3 males, mean age ± SD was 59.00 ± 3.62) and 8 age and sex matched healthy controls (4 males, mean age ± SD was 60.00 ± 3.46). Mean years of disease duration ± SD of patients was 6.86 ± 3.34, mean total UPDRS scores ± SD was 39.25 ± 22.64, mean UPDRS III score ± SD was 22.75 ± 17.17, mean H&Y score ± SD was 2.25 ± 0.65. Peripheral blood mononuclear cells (PBMCs) were isolated from whole blood by using Ficoll density gradient centrifugation and cryopreserved within FBS and 10% DMSO at liquid nitrogen until assayed. RNA was isolated from frozen PBMCs by using RNA isolation commercial kit (Jena Bioscience, Total RNA Purification Kit, PP-210L). Purity assessment was determined by O.D. ratios of 260/280 and 260/230 and concentrations of isolated RNAs were detected with Thermo Scientific Nanodrop 2000. cDNA was synthesized with a transcription kit (Jena Bioscience, PCR511) from 2 µl RNA with the concentration of 100 ng/ml by using Bio-Rad Thermal Cycler according to the manufacturer’s protocol. The quantitative real time PCR (qRT-PCR) assay was performed on an Agilent Stratagene 3005P system. Each reaction was run in 10 μl reaction mixture containing 1 μl of cDNA (50 ng/μl), 10 pmol/µl of forward and reverse primer sets for each specific gene (Table 1) by using Sybr green method (PCR master kit, Jena Bioscience, qPCR GreenMaster with UNG/lowROX kit, PCR306). Beta-actin (ACTB) was used as a normalization reference gene for quantitating the relative levels of gene expressions. Relative gene expressions were analyzed by 2^−ΔΔCt^ method^27^.

**Table 1:** Primers for real time PCR.

Gene	Forward primer	Reverse primer
CASP3	ATTGTGGAATTGATGCGTGA	GGCAGGCCTGAATAATGAAA
PRKACB	GCCACGACAGATTGGATTG	TCCAGAGCCTCTAAACTTTGGT
ACTB	AACCGCGAGAAGATGACCCA	GGATAGCACAGCCTGGATAGCA
GATA2	CCGCCACATCCATCCTAGCAG	TGCAGACGGCAACGGC
TSC2	AGCTCACGGAAACCTGTCTG	AGGAGACCTCTTCGGGACAG
PI3KCA	TCAAAGGATTGGGCACTTTT	GCCTCGACTTGCCTATTCAG

### Immunophenotyping by flow cytometry

To validate the findings of microarray study, a flow cytometry study was conducted in order to determine peripheral immune cell phenotype in PD patients. Frozen PBMCs obtained from 20 PD patients (mean age ± SD was 55.36 ± 5.41 mean age, 12 males) and 20 age and sex matched healthy controls (p = 0.111 and p = 0.744 respectively) from the original group (mean age ± SD 53.55 ± 7.63, 13 males) (8 PD and 8 controls were selected from subjects that were enrolled in RT-PCR study). Mean years of disease duration ± SD of patients was 7.17 ± 2.73, mean total UPDRS scores ± SD was 45.15 ± 19.04, mean UPDRS III score ± SD was 26.15 ± 14.63 mean H&Y score ± SD was 2.1 ± 0.55. Frozen PBMCs were used for extracellular labeling, anti-human monoclonal antibodies applied as followed: CD3-FITC, CD16/CD56-PE, CD45-PerCP, CD19-APC (Beckton Dickens (BD)-MultitestTM, Franklin Lakes, NJ, USA), CD3-Alexa Fluor 700, CD4-PerCP, CD8-APC-Cy7, CD25-APC, CD127-PE-Cy7, CD49d-PE-Cy5 (all from BD) and incubated for 20 min in the dark at room temperature. For intracellular IL-10 labeling at least 10^6^ PBMCs were incubated with 0.25 μg/ml Phorbol-12-myristate-13-acetate (PMA) (AdipoGen, San Diego, CA, USA), 1 μg/ml ionomycin (Santa Cruz, Dallas, TX, USA), and 10 μg/ml brefeldin (Ebioscience, Santa Clara, CA,USA) in complete medium (RPMI 1640 enriched with 10% fetal bovine serum, 1% nonessential amino acids, 1%L-glutamine, 1%Na-pyruvate, 1% minimum essential medium vitamin, 1% penicillin–streptomycin; Gibco, Waltham, MA, USA) for 16 h at 37 °C and %5 CO_2_. Then, PBMCs were permeabilized by 1 ml of fixation and permeabilization buffer (FoxP3 Staining Buffer Set, eBioscience) and incubated at 4 °C for an hour on ice. After washing with fixation buffer, PBMCs were stained with anti-IL-10-PE antibody (BD), and incubated at 4 °C for an hour on ice. Experiments were utilized in Novocyte (ACEA Bioscience, Inc.) and data were analyzed by using NovoExpress and Flowjo software. The results were finally expressed as percentage of positive cells of the parent (%).

### Statistical analyses

Categorical variables between groups were compared by chi-square test, Student *t* test was used for comparison of parametric variables and Mann–Whitney *U* for non-parametric variables. Pearson correlation was used to determine correlation between percentages of immune cell subtypes, expression levels of genes and clinical findings. A p value of < 0.05 was considered statistically significant. Statistical analyses were conducted using Prism (GraphPad Software, Inc) or SPSS software (IBM).

### Ethics approval and consent to participate

Written informed consent was obtained from all participants and study was approved by Istanbul University, Istanbul Faculty of Medicine Ethical Committee and conformed to the provisions of the Declaration of Helsinki.

## Results

Clinical features and demographical characteristics of subjects are presented in Table 2.

**Table 2:** Clinical and demographical characteristics of participants.

	PD (n = 30)	HC(n = 30)	p value
Gender (female/male)	10/20	11/19	0.518
Age (mean ± SD)	56.51 ± 1.40	51.18 ± 1.33	0.007
Age at disease onset, (mean ± SD)	49.36 ± 9.17		
Disease duration, years (mean ± SD)	6.91 ± 3.32		
**UPDRS total (mean ± SD)**	42.45 ± 19.02		
UPDRS I	6.57 ± 4.15		
UPDRS II	11.09 ± 8.36		
UPDRS III	23.57 ± 14.34		
UPDRS IV	1.85 ± 5.48		
H&Y scale	2.12 ± 0.60		

*H&Y Scale* Hoehn and Yahr Scale, *HC *healthy controls, *UPDRS* :Unified Parkinson's Disease Rating Scale, *PD* Parkinson’s disease.

### Analysis of DEGs

The boxplots for normalized gene expression data represent a good normalization (Supplementary Figure 1). After statistical assessment a total of 361 DEGs (143 upregulated and 218 downregulated) were identified between PD patients and controls (Supplementary Table .1). Hierarchical clustering and heat map analysis with 361 DEGs were performed for validation (Supplementary Figure 2).

### Functional annotation

GO analysis revealed that DEGs were involved in 28 biological processes, 12 cellular components and 26 molecular functions (Table 3).

**Table 3:** GO analysis of DEGs.

GO-ID	Term	Count	p value
**Biological process (BP)**			
GO:0006334	Nucleosome assembly	12	1.80E−05
GO:0006351	Transcription, DNA-templated	61	1.50E−04
GO:0043484	Regulation of RNA splicing	6	2.00E−04
GO:0008380	RNA splicing	12	2.00E−04
GO:0071456	Cellular response to hypoxia	8	0.002
GO:0048536	Spleen development	5	0.005
GO:0098609	Cell–cell adhesion	13	0.006
GO:0006397	Mrna processing	10	0.008
GO:0000122	Negative regulation of transcription from RNA polymerase II promoter	24	0.012
GO:0051028	mRNA transport	5	0.012
GO:0045814	Negative regulation of gene expression, epigenetic	5	0.015
GO:0045931	Positive regulation of mitotic cell cycle	4	0.016
GO:0045214	Sarcomere organization	4	0.018
GO:0043486	Histone exchange	3	0.018
GO:0060968	Regulation of gene silencing	3	0.018
GO:0006974	Cellular response to DNA damage stimulus	10	0.019
GO:0060325	Face morphogenesis	4	0.019
GO:0006278	RNA-dependent DNA biosynthetic process	3	0.021
GO:0045893	Positive regulation of transcription, DNA-templated	18	0.021
GO:0007160	Matrix adhesion	6	0.029
GO:0051726	Regulation of cell cycle	7	0.032
GO:0080182	Histone H3-K4 trimethylation	3	0.033
GO:0018105	Peptidyl-serine phosphorylation	7	0.033
GO:0000183	Chromatin silencing at rdna	4	0.034
GO:0018117	Protein adenylylation	2	0.038
GO:0036303	Lymph vessel morphogenesis	2	0.038
GO:0035574	Histone H4-K20 demethylation	2	0.038
GO:0043488	Regulation of mrna stability	6	0.048
**Cellular component (CC)**			
GO:0005654	Nucleoplasm	91	4.80E−08
GO:0005634	Nucleus	148	5.30E−08
GO:0000786	Nucleosome	9	3.70E−04
GO:0005737	Cytoplasm	125	8.70E−04
GO:0005720	Nuclear heterochromatin	4	0.008
GO:0005913	Cell–cell adherens junction	14	0.008
GO:0019013	Viral nucleocapsid	4	0.015
GO:0070062	Extracellular exosome	66	0.035
GO:0002944	Cyclin K-CDK12 complex	2	0.037
GO:0043234	Protein complex	14	0.045
GO:0005694	Chromosome	6	0.046
GO:0000788	Nuclear nucleosome	4	0.048
**Molecular function (MF)**			
GO:0044822	Poly(A) RNA binding	51	2.40E−08
GO:0005515	Protein binding	202	1.60E−04
GO:0042393	Histone binding	10	5.80E−04
GO:0016779	Nucleotidyltransferase activity	5	0.002
GO:0031492	Nucleosomal DNA binding	6	0.002
GO:0003677	DNA binding	50	0.002
GO:0003723	RNA binding	22	0.002
GO:0004674	Protein serine/threonine kinase activity	17	0.003
GO:0004693	Cyclin-dependent protein serine/threonine kinase activity	5	0.004
GO:0003720	Telomerase activity	3	0.010
GO:0031625	Ubiquitin protein ligase binding	13	0.010
GO:0098641	Cadherin binding involved in cell–cell adhesion	13	0.010
GO:0001968	Fibronectin binding	4	0.013
GO:0003714	Transcription corepressor activity	10	0.017
GO:0000166	Nucleotide binding	14	0.017
GO:0003682	Chromatin binding	15	0.019
GO:0004672	Protein kinase activity	14	0.022
GO:0003676	Nucleic acid binding	29	0.025
GO:0004864	Protein phosphatase inhibitor activity	4	0.025
GO:0005524	ATP binding	40	0.031
GO:0070733	Protein adenylyltransferase activity	2	0.038
GO:0035575	Histone demethylase activity (H4-K20 specific)	2	0.038
GO:0097493	Structural molecule activity conferring elasticity	2	0.038
GO:0003779	Actin binding	11	0.043
GO:0008307	Structural constituent of muscle	4	0.046
GO:0001046	Core promoter sequence-specific DNA binding	4	0.049

KEGG pathway analysis revealed that upregulated genes were mainly enriched in p53 (CASP3, TSC2, ATR, MDM4, CCNG1) and PI3K/Akt (IL2RA, IL4R, TSC2, VEGFA, PKN2, PIK3CA, ITGA4, BCL2L11) signaling pathways and downregulated genes were enriched in leukocyte transendothelial migration pathway (ACTG1, RAPGEF3, JAM3, MYL9, PTPN11) (Tables 4 and 5). Thus, several significantly altered genes were involved in survival and inflammation processes.

**Table 4:** Pathway analysis of upregulated genes in PD.

Category	Term	Genes	Count	p value
KEGG_PATHWAY	hsa04115:p53 signaling pathway	CASP3, TSC2, ATR, MDM4, CCNG1	5	0.0030
REACTOME_PATHWAY	R-HSA-211227:R-HSA-211227 (Activation of DNA fragmentation factor)	HIST1H1E, CASP3, HIST1H1D	3	0.0085
REACTOME_PATHWAY	R-HSA-1221632:R-HSA-1221632 (Meiotic synapsis)	SYNE2, HIST2H2BE, HIST2H2AC, SUN1, ATR	5	0.0107
REACTOME_PATHWAY	R-HSA-212436:R-HSA-212436 (Generic Transcription Pathway)	ZNF419, ZNF558, ZNF692, ZNF160, ZNF141, ZNF550, ZIK1, ZNF480, ZNF615, ZNF37A	10	0.0160
KEGG_PATHWAY	hsa04151:PI3K-Akt signaling pathway	IL2RA, IL4R, TSC2, VEGFA, PKN2, PIK3CA, ITGA4, BCL2L11	8	0.0338
KEGG_PATHWAY	hsa05414:Dilated cardiomyopathy	PRKACB, ITGA4, TPM2, TTN	4	0.0392
KEGG_PATHWAY	hsa05206:MicroRNAs in cancer	DNMT3A, CASP3, VEGFA, ZEB2, MDM4, CCNG1, BCL2L11	7	0.0416
REACTOME_PATHWAY	R-HSA-606279:R-HSA-606279 (Deposition of new CENPA-containing nucleosomes at the centromere)	HIST2H2BE, HIST2H2AC, CENPC, CENPT	4	0.0468
KEGG_PATHWAY	hsa03015:mRNA surveillance pathway	SMG5, SMG1, PABPC1L, NXT2	4	0.0479

**Table 5:** Pathway analysis of downregulated genes in PD.

Category	Term	Genes	Count	p-value
KEGG_PATHWAY	ptr04670:leukocyte transendothelial migration	ACTG1, RAPGEF3, JAM3, MYL9, PTPN11	5	0.0116

### PPI network construction

**Figure 1:**
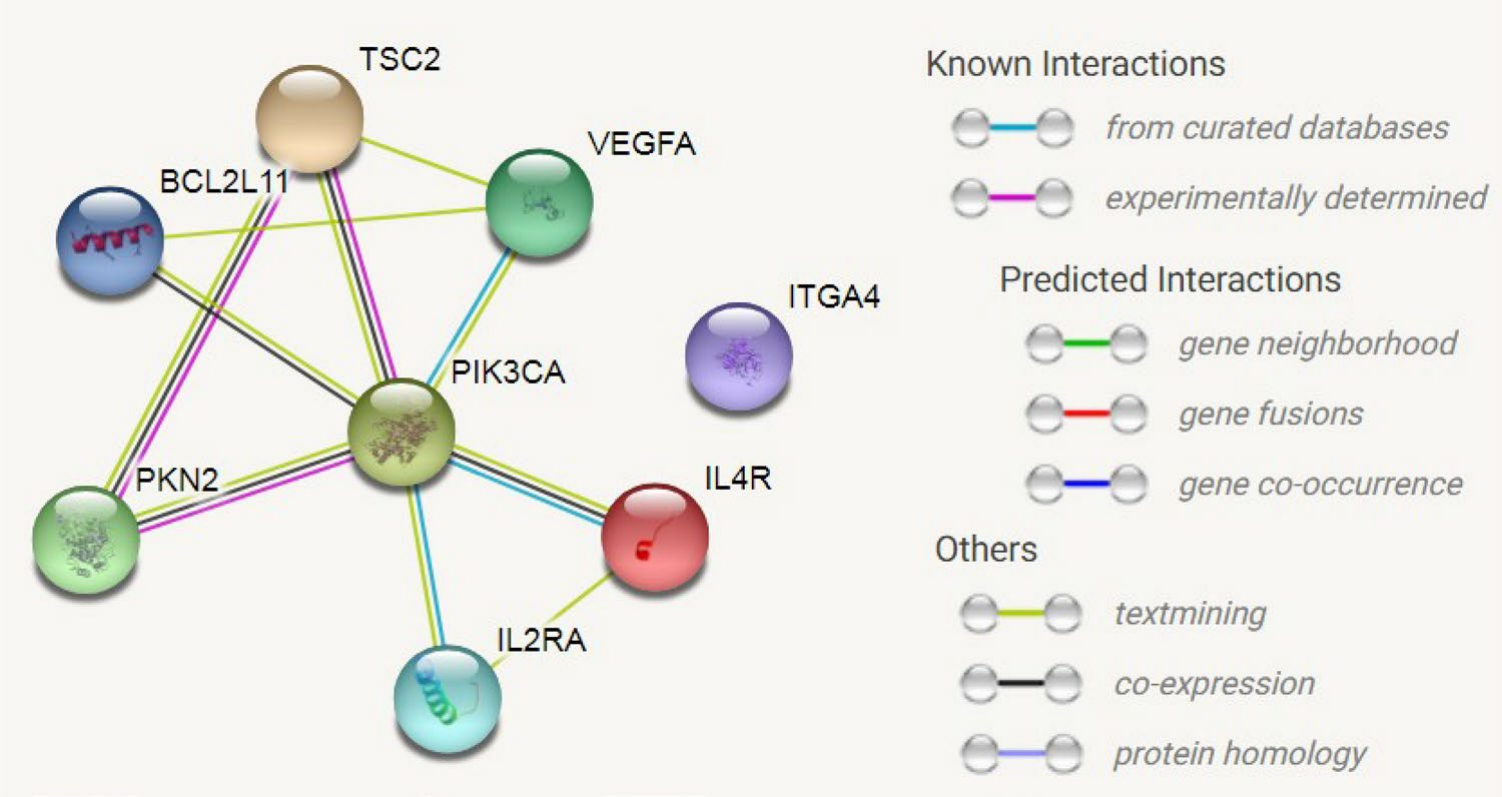
Protein–protein interaction (PPI) network for upregulated DEGs in PI3K/Akt pathway. Nodes represent proteins and lines represent interactions of proteins in networks.

**Figure 2:**
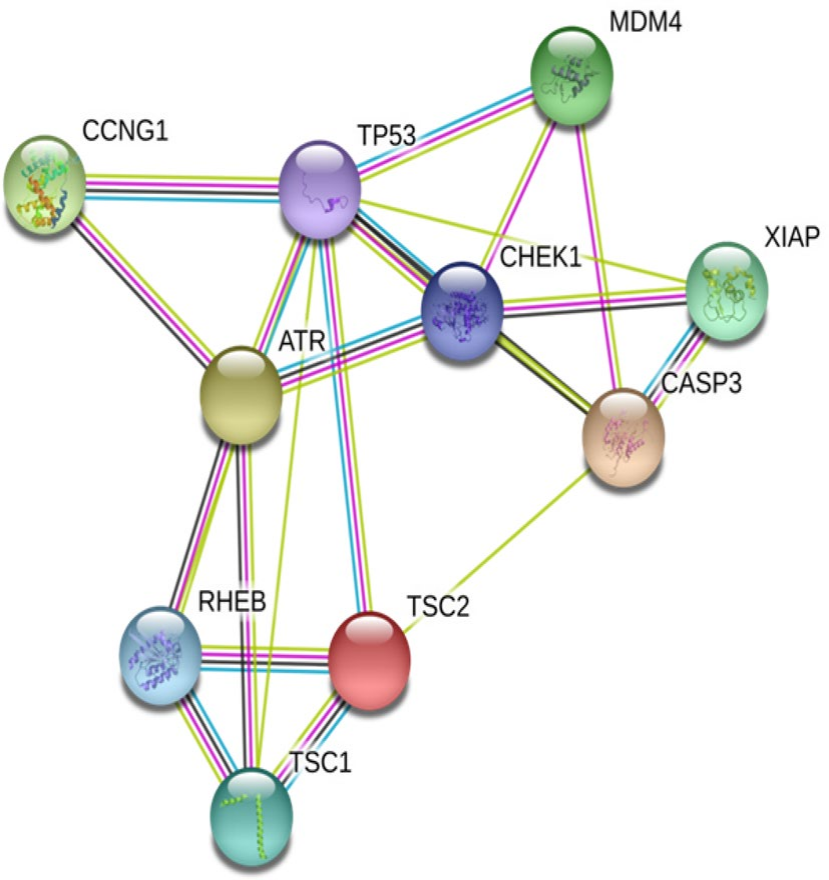
Protein–protein interaction (PPI) network for upregulated DEGs in p53 pathway. Nodes represent proteins and lines represent interactions of proteins in networks.

PPI network of proteins that were encoded by most significantly upregulated DEGs were involved in p53 and PI3K/Akt signaling pathways. TP53 and PIK3CA were identified to be most significant hub proteins (Figs. 1 and 2).

### Validation of gene expressions with RT-PCR

For validation of the microarray data, expression levels of 5 genes (PRKACB, TSC2, GATA2, PI3KCA, CASP3) that were randomly selected among the upregulated DEGs were quantified by RT-PCR analysis using peripheral blood samples of randomly selected 8 PD patients and 8 healthy controls. In consistency with microarray data, expression levels of selected genes were elevated in PD patients compared to healthy controls (Table 6). There was no correlation between expression levels of selected genes and clinical findings of PD patients such as age, age of disease onset, disease duration, UPDRS and H&Y scale scores (data not shown).

**Table 6:** Fold chance values of RT-PCR and microarray data.

	Q-RT-PCR FC*	Microarray FC
PRKACB	1.951	1.725
TSC2	1.191	1.615
GATA2	1.042	1.657
PI3KCA	2.188	1.156
CASP3	1.579	1.348

*Q-RT-PCR* quantitative real time polymerase chain reaction, *FC* fold change.

*Note that FC was assessed by the 2^−ΔΔCt^ method for Q-RT-PCR.

### Flow cytometry analyses

**Figure 3:**
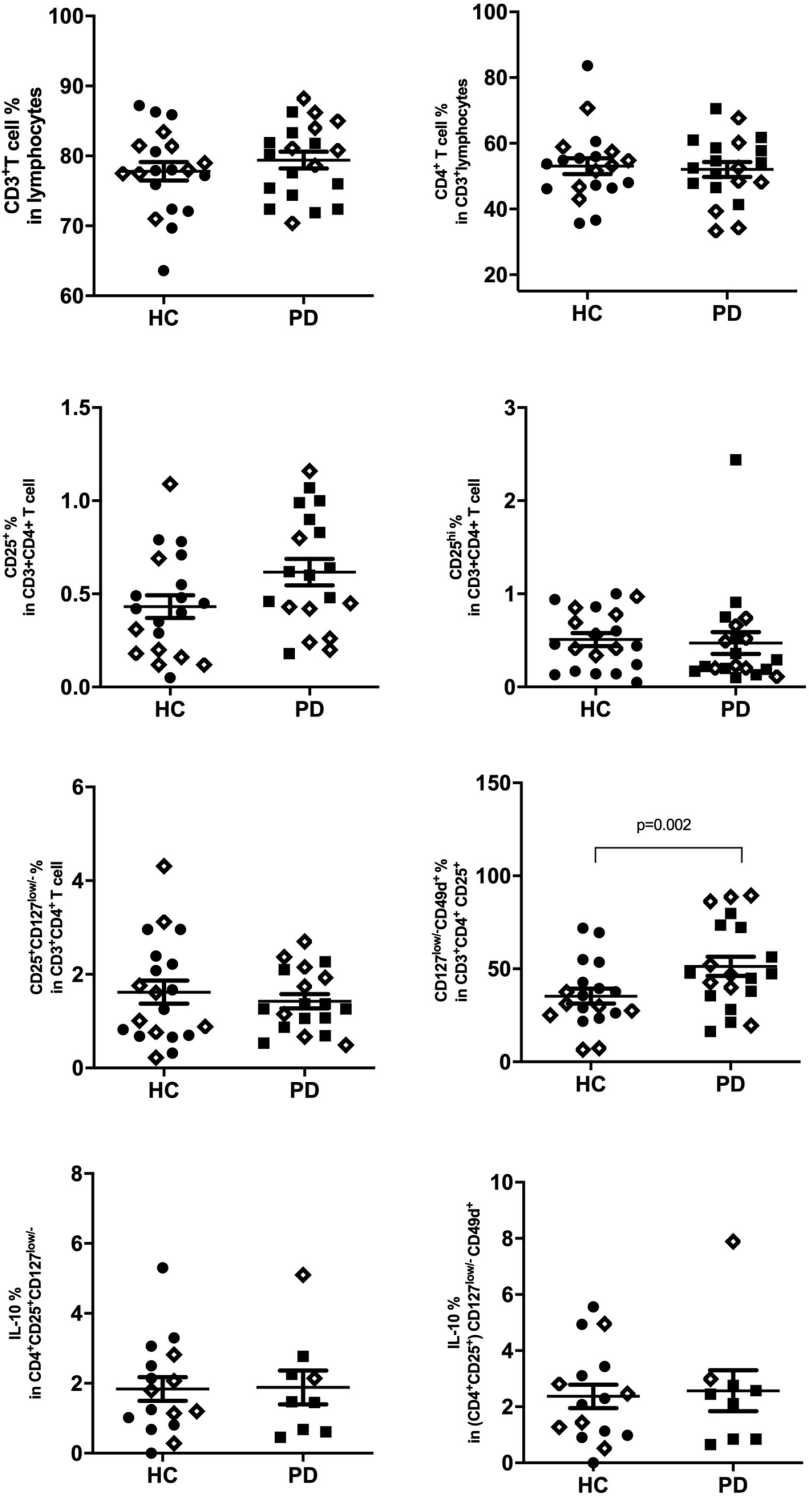
Frequencies of T cell subsets are shown on y axis for PD and control groups, symbol ◊ represents subjects enrolled in both RT-PCR and flow cytometry studies.

**Figure 4:**
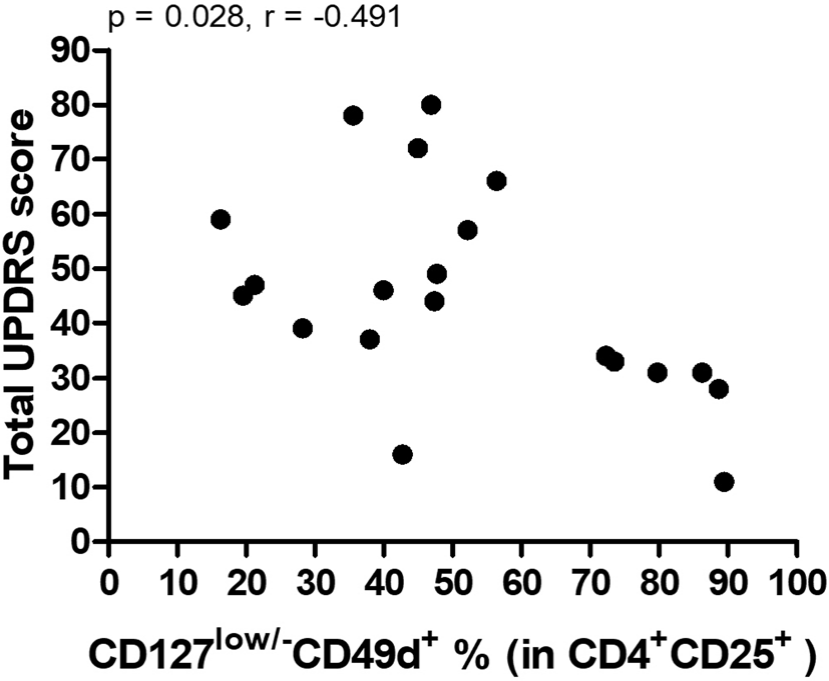
Correlation plot between total UPDRS score and proportion of CD4^+^CD25^+^CD127^low^CD49d^+^ Tregs.

An important finding of the microarray data was enhanced expression of Treg-associated surface expression factors IL2RA (CD25) and CD49d (Suppl. Table 1). To validate the alteration of Treg populations in PD, peripheral blood frequencies of immune cell subsets were assessed by flow cytometry. The gating strategy has been displayed in Supplementary Figure 3. Frequencies of CD3^+^ T cells (79.40 ± 1.201 vs. 77.81 ± 1.331), CD4^+^ helper T cells (52.06 ± 2.259 vs. 53.05 ± 2.430), CD8^+^ cytotoxic T cells (33.57 ± 1.875 vs. 31.39 ± 1.932), CD19^+^ B cells (5.805 ± 0.4360 vs. 5.651 ± 0.5065) and CD16/56^+^ NK cells (11.06 ± 1.17 vs. 14.31 ± 1.12) were comparable between PD and control groups. Percentage of CD3^+^CD4^+^CD25^+^ T cells and CD3^+^CD8^+^CD25^+^ T cells were slightly increased in PD group without attaining statistical significance. Frequency of Tregs, which were defined as CD4^+^CD25^+^CD127^low^ T cells^28^, showed no differences among patient and control groups. For further evaluation of effects of CD49 expression on suppressive capacity of Tregs, subpopulations of CD49d positive and negative cells were evaluated within CD4^+^CD25^+^CD127^low^ Tregs. Frequency of CD49d^+^ Tregs were significantly higher in PD patients than in controls (51.33 ± 5.187 vs 35.33 ± 4.035, p = 0.002). To assess functional immunosuppressive capacity of Tregs, we compared frequencies of IL-10 producing cells within CD4^+^CD25^+^CD127^low^ and CD4^+^CD25^+^CD127^low/-^CD49d^+^ subtypes between PD and controls. There were no differences between two groups in terms of frequencies of IL-10 producing cells for both subtypes (Fig. 3). Proportions of CD4^+^CD25^+^CD127^low^CD49d^+^ Tregs were found to be negatively correlated with total UPDRS score in PD patients (p = 0.028, r = -0.491) (Fig. 4). There was no correlation between CD49d^+^ Treg frequencies versus age or disease duration.

## Discussion

PD is an important health care problem that causes serious impairment in daily life quality especially in elderly population. The disease can only be treated symptomatically with the current treatment strategies and the degenerative process cannot be prevented. An important reason for this is that significant loss of neurons has already occurred at the stage of diagnosis. Improved understanding of the pathophysiology of PD and the mechanisms underlying neuronal loss are crucial for early diagnosis and development of disease-specific treatments. Our results revealed that genes that are most differentially expressed between PD patients and healthy controls are particularly associated with survival pathways and inflammation. p53 and PI3K/Akt pathways were the most profoundly affected survival-associated pathways.

Apoptosis is crucially involved in physiological and pathological conditions such as oxidative stress and severe DNA damage. Genotoxic stress in the cell is recognized by ataxia telangiectasia mutated (ATM) kinase and ATM- and Rad3-related (ATR) kinase proteins, which provide phosphorylation of murine double minute gene 2 (MDM2), a ubiquitin-ligase that enhances p53 degradation^29,30^. This posttranslational change causes inactivation of MDM2 while activating p53^31^. Activated p53 stops the cell cycle in order to eliminate DNA damage. In cases that the DNA damage cannot be corrected, p53 initiates programmed cell death. p53-induced pro-apoptotic genes cause release of cytochrome-*c* from the mitochondrial membrane. Cytochrome-*c* also interacts with p53-induced APAF-1 protein to activate the caspase cascade^32^. p53 suppresses the Akt/mTOR pathway both by enhancing tuberous sclerosis complex 2 (TSC2) transcription and inducing synthesis of PTEN protein that degrades PIP3^33,34^. In postmortem studies and experimental models of PD, apoptosis has been shown as an important mediator of dopaminergic cell death^35^. p53, an important tumor suppressor gene, plays a critical role in the process of apoptosis by transcriptional control of the genes associated with cell death and Bcl2 family interaction^36^. SNpc of PD brains shows increased p53 expression^37^, deletion of DJ1 causes p53-mediated loss of dopaminergic neurons in experimental models, p53 inhibition has a neuroprotective effect and p53 activates transcription of the alpha synuclein gene (33–34). Thus, overexpression of the p53 pathway might be one of the essential factors involved in the pathogenesis of sporadic PD.

PI3K-Akt signaling pathway is a highly conserved cascade that promotes cell survival and proliferation and plays important role in synaptic plasticity. Numerous growth factors and cytokines carry out their intracellular effects via PI3K pathway^38^. In our study, PPI analysis showed that phosphatidylinositol-4,5-bisphosphate 3-kinase catalytic subunit alpha (PI3KCA) protein has a central role in this network. The PI3K enzyme catalyzes the synthesis of an important secondary messenger phosphatidylinositol (3,4,5)-trisphosphate (PIP3) molecule from the phosphatidylinositol 4,5-bisphosphate (PIP2). PIP3 activates phosphorylation of the serine threonine kinase Akt^39^. mTORC1, one of the downstream proteins of this signaling pathway, is a protein kinase complex that acts as a sensor for metabolic status of the cell and plays an important role in decision-making in the direction of survival or apoptosis^40^. Akt eliminates inhibition of mTORC1 by phosphorylating the TSC2 protein, which has an inhibitory effect on mTORC1. Activation of mTORC1 increases anabolic activity in the cell and suppresses autophagy. Defects in this pathway have been associated with neurodegeneration in diseases such as Alzheimer and Huntington disease^40–42^. In an experimental study, TSC2 expression was shown to be increased in response to synuclein accumulation in the transgenic mouse model, but no significant difference was found between TSC2 expression levels in the brain of human PD cases and controls^43^. In another study, it has been shown that proteins regulating autophagy prevent the ubiquitination of TSC2 in the neurotoxin-induced animal model and cause autophagy in dopaminergic neurons through this mechanism^44^. Activation of autophagic mechanisms may protect dopaminergic neurons from synuclein toxicity by increasing synuclein degradation^45^. In our study TSC2 gene expression was increased in the PD group and this upregulation might be due to the effort to activate the autophagy process and could be a therapeutic target, especially in the early stage of the disease.

Other members of the PI3K pathway that are upregulated in PD patients are also profoundly associated with the disease. Akt regulates the expression of Bcl-2 like 11 (BCL2L11) through phosphorylation of forkhead phosphorylation of transcription factors (FOXO)^46^. BCL2L11, also known as Bim (Bcl 2-interacting mediator of cell death), is a potent pro-apoptotic protein that can bind anti-apoptotic proteins with high affinity^47^. In the MPTP-induced PD mouse model, Bim expression is increased regionally in the midbrain and remains high during dopaminergic neuron death^48^. VEGFA is the biologically most active isoform of VEGF growth factor family that potently promotes proliferation and migration of vascular endothelial cells and controls vascular permeability ^49,50^. Although VEGFA is primarily identified as an angiogenesis factor, recent studies have implied neurotrophic, neuroprotective and chemoattractant roles, as well^51^. Upregulated expression of VEGF gene has been reported in reactive astrocytes within substantia nigra of PD patients^52^. This upregulation is likely to have occurred as a compensatory mechanism to enhance neurogenesis and to maintain microcirculation.

In this study, we found increased expression levels of several inflammation-related genes. Proteomics analysis suggested that upregulation of at least some of the inflammation genes (e.g. IL-2RA and IL-4R) was by virtue of the enhanced expression of the PI3K/Akt pathway, which is intimately associated with inflammatory pathways. Early evidence of involvement of inflammation in PD pathogenesis has come from the studies that revealed presence of human leukocyte antigen DR (HLA-DR) positive reactive microglia in SNc of PD patients. Subsequent studies showed that increased expression of proinflammatory cytokines released from activated microglia contributes to the degeneration of dopaminergic neurons and activation of nuclear factor kappa B (NF-κB) pathway in PD patients and experimental models of PD^53–57^. Consistent with these findings increased expression of genes encoding proinflammatory cytokines has been found in SN of PD patients^58^.

Inflammatory factors may display both hazardous and neuroprotective roles in PD. As an example, IL-4, an anti-inflammatory cytokine, may both suppress microglial activity and modulate neurogenesis^59,60^. In the lipopolysaccharide-treated rat model, increased endogenous IL-4 synthesis caused elevation of pro-inflammatory cytokines of microglial origin thereby contributing to degeneration of vulnerable dopaminergic neurons^61^. By contrast, infiltrating IL-4 producing CD4^+^ T cells in the CNS have been shown to improve neuronal survival and achieve axonal healing through the Akt signaling pathway^62^. Similarly, in experimental autoimmune encephalitis models, externally administered IL-4 has been shown to improve axonal damage via IL-4 receptor (IL-4R)-mediated signaling pathway^63^. While PD is typified with Th1-type inflammation^64^, IL-4 may shift the balance in favor of Th2-type inflammation and thus may regulate neuroinflammation in PD. IL-4 also prevents neuronal death and induces neuronal growth through binding IL-4 receptor alpha (IL4-Rα) on neurons^62^. Thus overexpression of IL4-R in PD might be a compensating countermeasure to suppress the ongoing neuroinflammation.

Although the role of the peripheral immune system in PD-related neuroinflammation has not yet been entirely elucidated, infiltrating CD4^+^ and CD8^+^ T lymphocytes have been suggested to contribute to neurodegeneration^65^. CD3^+^ T cells, which have been found in close proximity to glial cells in brains of DLB patients and α-synuclein transgenic mice, have been suggested to participate in activation of glial cells^66^. In vitro studies suggested that CD4^+^/CD25^−^ effector T cells promote microglial activation and contribute to neurodegeneration while CD4^+^/CD25^+^ Tregs might be inhibiting microgliosis^67^. Furthermore, adoptive transfer of CD3 activated Tregs to mice with MPTP-induced PD has provided greater than 90% protection of the nigrostriatal system^68^. It is thought that dysfunction of Tregs may contribute to PD pathogenesis by enhancement of neuroinflammation or by impairment of tolerance to neuronal antigens^69^. However, the underlying mechanisms of decreased effectiveness of regulatory T cells in PD patients have not been elucidated.

IL2RA (CD25) gene encodes the alpha chain of the receptor complex of IL-2, an important cytokine associated with immune homeostasis and self-tolerance. IL-2 acts as a growth factor for T cells^70^. CD25^+^ Tregs suppress the inflammatory response and autoimmunity presumably by competing for and consuming the IL-2 cytokine. The effector CD4^+^ T cell apoptosis induced by Treg cells through cytokine deprivation has been shown to require the presence of BIM proapoptotic protein^71^, which was upregulated in our study. Dopaminergic cell damage induced by 1-methyl-4-phenylpyridinium (MPP+) is prevented by Tregs. Suppression of microglial oxidative stress and inflammation by Tregs might likely be responsible for this protective mechanism.

There are controversial results for proportion of Tregs in PD. While in some studies Tregs were found to be increased in PD, some other researchers reported that Tregs were decreased or not altered in PD patients^72–74^. Saunder et al. found that Tregs from PD patients had reduced ability to suppress effector T cells while proliferative capacity of CD4^+^ T cells did not change. Besides, α4β1^+^ CD4^+^ T cells were elevated slightly but not significantly in PD patients^75^. α4β1 integrin is an adhesion molecule that facilitates the migration of immune system cells into the central nervous system. The alpha chain of α4β1 integrin is encoded by ITGA4 (CD49d) gene. The ability of CD49d^+^ Tregs to suppress effector T cells was found to be weaker compared to CD49d negative T cells in vitro^76^. In our study, CD49d expression was increased in PD and also PD patients displayed higher frequencies of CD49d^+^ Tregs in the peripheral blood. Notably, PD patients with higher frequencies of CD49d^+^ Tregs showed trends towards displaying lower UPDRS scores, indicating a neuroprotective role of this Treg subset in PD presumably through suppression of infiltrating T cells in the brain. These results could also be explained by reduction of CD49d^+^ Tregs by increased age and disease duration. However, no correlation could be found between these factors ruling out this assumption.

It is known that neuroinflammation induced by activation of astrocytes and microglia with subsequent dysfunction of endotelhial cells contributes to PD pathogenesis. Besides central inflammation, peripheral proinflammatory profile with altered CD4^+^/CD8^+^ T cells ratios and Treg cells might facilitate neurodegeneration^77^. Several transcriptome studies that were performed in different platforms with different tissues have highlighted the importance of inflammatory pathways in PD pathogenesis^78^. These studies have also shown that even different regions of PD brain may show altered immune gene expression profiles^79^. Highly diverse DEG profiles have been obtained in previous transcriptome studies that were recently conducted with peripheral blood samples of sporadic PD patients with a special emphasis on immune system-related genes (Table 7)^16,18,19,23,81–93^. Possible reasons underlying this variability could be the heterogeneous physiopathology of sporadic PD, different inclusion criteria and clinical confounders (such as early onset, treatment status and exercise), different expression detection methods (microarray or RNA-seq) and diverse enrichment analysis methods. In general, expression levels of inflammatory factors are increased in the peripheral blood of PD patients. Nevertheless, essential T lymphocyte activation genes have also been found to be decreased in the reports of Jiang et al., Chi et al. and Calligaris et al.^16,83,84^. It is tempting to speculate that this may be attributed to the increased Treg activity, which was demonstrated by our study. In addition, Calligaris et al. and Mutez et al. reported an alteration in the signaling pathway of IL-4R (a receptor highly abundant on Tregs), while we found altered expression of the IL-4R^16,85^. The fact that the recruited patients in the Calligaris study were not under treatment, unlike our patient cohort, suggests that the altered expression of this pathway cannot be attributed to dopaminergic therapy. Another resemblance with our findings was altered expression of cytokine receptor (e.g. IL-18R, IL-2RA) and cell adhesion molecule (e.g. ICAM1) gene expression levels in studies reported by Kurvits et al., Hu et al. and Tan et al.^19,81,82^. Furthermore, altered expression levels of Treg related genes were highlighted by Mutez et al. and Pinho et al.^85,93^. Grünblatt et al. reported increased expression of integrin, alpha M (ITGAM) in substantia nigra of sporadic PD patients^80^ By contrast, immune-related genes that were significantly altered in our study and not reported in previous studies were VEGFA and ITGA4.

**Table 7:** Comparison of the recently performed peripheral blood transcriptome studies of PD patients with our study.

Authors	Patient selection criteria	Method	Enrichment analyses	Number of samples	Most significant DEGs related with immune system	Most significant pathways related with immune system	Comment
Kurvits et al.^81^	Idiopathic PD with standard medical treatment	Whole blood RNA-Seq	KEGG	12 PD, 12 HC	IL18R1 (upregulated)	Immune system related pathways were not specified	IL18R1 is an interleukin receptor linked with proinflammatory responses
Hu et al.^82^	Idiopathic PD without any immune system disorders and under standard medical treatment	RNA-Seq from blood leukocytes before and after exercise	KEGG	21 PD	CCL5, CD28, CD3E, IL23A, IL2RA, PRKCQ, TMIGD2, TNFSF9, TNFRSF18 ZAP70 (downregulated after exercise)	T-cell receptor signaling pathway, cytokine receptor-signaling pathway and primary immunodeficiency pathway were highly enriched in downregulated genes after exercise	Exercise can modulate the abnormal immunity in patients with PD through T-cell-related functions
Jiang et al.^83^	Early-stage idiopathic PD	Microarray (Affymetrix)	KEGG, CYTOSCAPE	255 PD, 255 HC	TNFSF14 (Upregulated), IL23A (Downregulated)	TNFSF14 was enriched in the pathways associated with immunoregulation	TNFSF14 stimulates the proliferation of T cells. IL23A is involved in Th17-type immune response
Tan et al.^19^	Drug-naïve sporadic PD	Microarray (Affymetrix)	KEGG, STRING	40 PD, 19 HC	CD40, GATA3 (upregulated) ICAM1 (downregulated)	Chemokine signaling pathway, Cytokine–cytokine receptor interaction, Cell adhesion molecules, NF-kappa B signaling pathway	ICAM1, other cell adhesion molecules and chemokines are involved in persistent neuroinflammation. GATA3 is a regulator of T-cell development
Chi et al.^84^	Early-stage idiopathic PD	Microarray (Affymetrix)	KEGG	50 PD, 22 HC	HLADQB1, IFI27 (downregulated)	The downregulated DEGs were enriched in the immune response	HLADQB1 is related with T cell activation
Calligaris et al.^16^	Drug-naïve sporadic PD	Microarray (Affymetrix)	GO, DAVID, GSEA	40 PD, 20 HC	TCF3, DOCK10, MAN1C1 (downregulated)	Lymphocyte activation, leukocyte activation	TCF3 is involved in development and differentiation of B and T cells. DOCK10 is involved in IL-4 receptor signaling. MAN1C1 is a key protein required for innate immunity activation
Mutez et al.^85^	Familial PD with LRRK2 mutation	Microarray (Agilent)	Ingenuity Pathways Analysis	10 familial PD, 7 HC, 1 pooled HC	Immune related genes were not specified	TGF-β signaling, IL-10 signaling, IL-4 signaling, leukocyte extravasation signaling, toll-like receptor signaling, IL-6 signaling	CD4^+^CD25^+^Foxp3 regulatory T cells modulate microglial inflammation through IL-10- and TGF-β-mediated mechanisms
Infante et al.^86^	PD with LRRK2 mutation and idiopathic PD	RNA-Seq	KEGG	20 familial PD, 20 idiopathic PD, 20 HC	Immune related genes were not specified	Complement cascade, cell adhesion molecules	The complement system is an effector arm of the innate immune response, which may play role in PD pathogenesis
Infante et al.^18^	PD with LRRK2 mutation and idiopathic PD	RNA-Seq	Not performed	20 familial PD, 20 asymptomatic carriers of the mutation, 20 idiopathic PD, 20 controls, 7 PD before and after initiating dopaminergic therapy	FCER2 (downregulated in PD)	Not specified	FCER2 is a key molecule for B-cell activation and growth and is involved in oxidative stress mediated damage
Soreq et al.^23^	Early stage PD	Microarray (Affymetrix)	BiNGO	50 PD, 22 HC, 33 NC	CR1, FCGRT CLEC4E, IGSF6, interleukins and their receptors, MEFV, IRF4 upregulated compared to HC; TNFSF14, TNFRSF9, TNFRSF10C upregulated compared to NC	IL-4 biosynthesis	Transcription and immune signaling-related transcripts are distinctly expressed in nucleated blood cells early in PD progress
Soreq et al.^87^	PD patients pre- and post-deep brain stimulation	Exon array	Post-hoc GO analyses	7 PD pre-DBS, 7 PD post-DBS (stim-ON), 7 PD post -DBS (stim-OFF), 6 HC	Immune related genes were not specified	IL-8 receptor activity, IL-1 receptor activity, Th1 and Th2 immune response, B cell differentiation, cytokine production	The DBS stimulus induced more leucocyte transcriptional change than the disease itself. This was reversed by DBS stimulation
Soreq et al.^88^	Advanced PD	Exon array	Matlab	7 PD, 6 HC	Immune related genes were not specified	NF-B cascade and immune response	PD patients’ blood leukocytes exhibit alternative splicing of immune response related genes
Alieva et al.^89^	Untreated PD at early stage	Microarray (pooled RNA) Illumina Human HT-12 v4.0	DAVID	4 PD, 4 HC	Immune related genes were not specified	Immune system process, Defense response, Response to cytokine stimulus, Positive regulation of I-kappaB kinase/NF-kappaB signaling	Immune system activation may play an important role in the development of PD
Karlsson et al.^90^	Patients with clinically defined PD, patients with de novo PD	Microarray Illumina Human HT-12 v4.0	Ingenuity Pathway Analysis	79 PD,75 HC	Immune related genes were not specified	Inflammatory response pathways	Changes in gene expression in the brain due to PD can also be found in blood and could be used as molecular biomarker for early detection of PD
Kobo et al.^91^	PD patients with GBA mutation	Exon array, Affymetrix	DAVID	59 PD with GBA mutation, 59 HC	BANK1, CD180, CD19, CD22, CD72, CD791, FCRL1, FCRL2, FCRL3,IGHD, IGHM downregulated	Immune response, B cell receptor signaling pathway	The down-regulation of B cell-related genes are observed in PD-GBA patients and correlate with disease progression
Li et al.^92^	Early-stage idiopathic PD	Microarray, Affymetrix	Ingenuity pathways analysis	50 PD, 22 HC	Immune related genes were not specified	T cell receptor signaling, iCOS-iCOSL signaling in T helper cells, CD28 signaling in T helper cells, PI3K signaling in B lymphocytes, B cell receptor signaling	mTOR signaling and CD28 signaling in T helper cells were able to accurately classify PD and healthy control samples
Pinho et al.^93^	PD with slow and rapid progression	Microarray, Affymetrix	DAVID	35 PD with slow progression and 35 PD with rapid progression	FOXP1 downregulated in rapid progressive patients	Immune function pathways	Decreased FOXP1 expression may result in increased levels of pro-inflammatory mediators
Karaaslan et al	Idiopathic PD with standard medical treatment	Microarray (Agilent)	KEGG, STRING	18 PD, 18 HC	IL2RA, IL4R, ITGA4 (CD49d)	Immune system related pathways were not specified	Upragulated genes were associated with Treg function, increased expression of ITGA4 (CD49d) might be the explanation of dysregulated Treg functions in PD

*PD* Parkinson’s disease, *HC* healthy control, *NC* neurological control, *BINGO* biological networks gene oncology, *KEGG* Kyoto Encyclopedia of Genes and Genomes, *DAVI*: database for annotation, visualization and integrated discovery, *STRING* search tool for the retrieval of interacting genes/proteins, *GO* gene ontology, *GBA* glucosidase beta acid.

To further understand the mechanism, by which CD49d^+^ Tregs might be suppressing neuroinflammation in PD, we assessed Tregs with anti-inflammatory IL-10 production and could not find difference between PD patients and controls. Nevertheless, Tregs might be operating through TGF-β rather than IL-10 production in PD and this assertion should be further investigated in future studies. Another limitation of our study in this context was not assessing pro-inflammatory cytokine-producing T cell subtypes and thus the inflammation status of our PD patients and suppression capacity of CD49d^+^ Tregs could not be duly investigated. Other limitations of our study were the low sample size for both gene expression analyses and validation studies. Although there were numerous gene expression studies with larger cohorts that revealed possible role of immune system in the pathogenesis of PD, the data we have obtained with a limited number of samples may provide a basis for discussing Treg-PD relationship in functional terms.

## Conclusion

In brief, we detected altered expression levels of genes involved in survival, apoptosis and inflammation related pathways in PD patients. We also found clues indicating the functionality of a subset of Tregs. Our results emphasize once again the complex interactions between neuronal apoptosis and inflammation in neurodegeneration. The exaggerated proinflammatory responses and/or insufficient anti-inflammatory mechanisms may result in the loss of vulnerable dopaminergic neurons. The increased detection of CD49d expression, which is expected to be low in Tregs with high suppressive capacity, suggested that dysfunctional Tregs may participate in PD pathogenesis. Nevertheless, increased CD49 expression may be a factor that positively affects the prognosis of the disease in the advanced stages through as yet unknown mechanisms.

## Supplementary Information

Supplementary Figures.

Supplementary Table 1.

## Data Availability

All data generated or analysed during this study are included in this published article and its supplementary information files.

## Abbreviations

DEGs: Differentially expressed genes
PD: Parkinson’s disease
RT-PCR: Real time polymerase chain reaction
Tregs: Regulatory T cells
SNpc: Substantia nigra pars compacta
UPDRS: Unified Parkinson disease rating scale
H&Y Scale: Hoehn and Yahr scale
GO: Gene ontology
KEGG: Kyoto Encyclopedia of Genes and Genomes
DAVID: Database for annotation, visualization and integrated discovery
STRING: Search tool for the retrieval of interacting genes/proteins
ATM: Ataxia telangiectasia mutated
ATR: ATM- and Rad3-related (ATR)
MDM2: Murine double minute gene 2
PI3KCA: Phosphatidylinositol-4,5-bisphosphate 3-kinase catalytic subunit alpha
PIP3: Phosphatidylinositol (3,4,5)-trisphosphate
PIP2: Phosphatidylinositol 4,5-bisphosphate
TSC2: Tuberous sclerosis complex 2
BCL2L11: Bcl-2 like 11
FOXO: Forkhead phosphorylation of transcription factors
MPP +: 1-Methyl-4-phenylpyridinium
