# Supplementary material for: Inflammation and regulatory T cell genes are differentially expressed in peripheral blood mononuclear cells of Parkinson’s disease patients
Source: Sci Rep. 2021 Jan 27;11:2316. doi: 10.1038/s41598-021-81961-7 (PMC7841172; doi:10.1038/s41598-021-81961-7)
Supplement: Supplementary file 1 — Supplementary Figures. [file 41598_2021_81961_MOESM1_ESM.docx]

Identification of Molecular Mechanisms in Parkinson’s Disease Pathogenesis: Significance of Survival and Inflammation Pathways

**Zerrin Karaaslan^1^, Özlem Timirci Kahraman^2^, Elif Şanlı^1^, Hayriye Arzu Ergen^2^, Başar Bilgiç^3^, Vuslat Yılmaz^1^, Erdem Tüzün^1^, Haşmet Ayhan Hanağası^3^,** **Cem İsmail Küçükali^1*^**

1. Department of Neuroscience, Aziz Sancar Institute of Experimental Medicine, Istanbul University, Istanbul, Turkey
2. Department of Molecular Medicine, Aziz Sancar Institute of Experimental Medicine, Istanbul University, Istanbul, Turkey.
3. Department of Neurology, Istanbul Faculty of Medicine, Istanbul University, Istanbul, Turkey.

***Corresponding author**: Cem İsmail Küçükali, MD, PhD, Istanbul University, Aziz Sancar Institute of Experimental Medicine, Department of Neuroscience, Istanbul,Turkey, [cemsmile@gmail.com](mailto:cemsmile@gmail.com)

E mail addresses in order: [zkaraaslan@gmail.com](mailto:zkaraaslan@gmail.com)

[ozlemtim@gmail.com](mailto:ozlemtim@gmail.com)

[elifsanli12@gmail.com](mailto:elifsanli12@gmail.com)

[aergen@istanbul.edu.tr](mailto:aergen@istanbul.edu.tr)

[bilgicb@gmail.com](mailto:bilgicb@gmail.com)

[vuslaty@hotmail.com](mailto:vuslaty@hotmail.com)

[drerdem@yahoo.com](mailto:drerdem@yahoo.com)

[hasmet@yahoo.com](mailto:hasmet@yahoo.com)

[cemsmile@gmail.com](mailto:cemsmile@gmail.com)

**Supplementary Table 1**: List of differentially expressed genes (DEGs) between Parkinson’s disease patients and control subjects

| **EntrezGeneID** | **Description** | **Log 2 Fold Change** | **Regulation** | **p-value** |
| --- | --- | --- | --- | --- |
| 23461 | ATP-binding cassette, sub-family A (ABC1), member 5 | 1,148 | up | 1,78E-08 |
| 11093 | Homo sapiens ADAM metallopeptidase with thrombospondin type 1 motif, 13 | 1,247 | up | 7,32E-08 |
| 4299 | Homo sapiens AF4/FMR2 family, member 1 | 1,182 | up | 3,13E-11 |
| 642517 | Homo sapiens ArfGAP with GTPase domain, ankyrin repeat and PH domain 9 | 1,020 | up | 4,17E-08 |
| 79026 | Homo sapiens AHNAK nucleoprotein | 1,012 | up | 3,46E-08 |
| 8852 | Homo sapiens A kinase (PRKA) anchor protein 4 | 1,751 | up | 8,23E-06 |
| 80709 | Homo sapiens AT-hook transcription factor | 1,042 | up | 4,00E-11 |
| 375248 | Homo sapiens ankyrin repeat domain 36 | 1,470 | up | 1,57E-06 |
| 8539 | Homo sapiens apoptosis inhibitor 5 | 1,010 | up | 3,35E-10 |
| 55082 | Homo sapiens arginine and glutamate rich 1 | 1,439 | up | 1,02E-08 |
| 445328 | Homo sapiens Rho guanine nucleotide exchange factor (GEF) 35 | 1,681 | up | 2,44E-04 |
| 100506084 | Homo sapiens ADP-ribosylation factor-like 17B | 1,347 | up | 1,66E-08 |
| 10973 | Homo sapiens activating signal cointegrator 1 complex subunit 3 | 1,071 | up | 4,32E-11 |
| 55729 | Homo sapiens activating transcription factor 7 interacting protein | 1,043 | up | 4,77E-11 |
| 64225 | Homo sapiens atlastin GTPase 2 | 1,043 | up | 6,17E-13 |
| 545 | Homo sapiens ATR serine/threonine kinase | 1,407 | up | 7,36E-15 |
| 4287 | Homo sapiens ataxin 3 | 1,175 | up | 2,64E-09 |
| 135152 | Homo sapiens beta-1,3-glucuronyltransferase 2 | 1,188 | up | 4,36E-08 |
| 10018 | Homo sapiens BCL2-like 11 (apoptosis facilitator) | 1,043 | up | 6,25E-07 |
| 9774 | Homo sapiens BCL2-associated transcription factor 1 | 1,045 | up | 1,52E-10 |
| 8019 | Homo sapiens bromodomain containing 3 | 1,110 | up | 6,27E-12 |
| 54014 | Homo sapiens bromodomain and WD repeat domain containing 1 | 1,068 | up | 5,16E-10 |
| 10438 | Homo sapiens C1D nuclear receptor corepressor | 1,011 | up | 7,88E-11 |
| 81617 | Homo sapiens calcium binding protein 39-like | 1,211 | up | 6,20E-08 |
| 57082 | Homo sapiens cancer susceptibility candidate 5 | 1,351 | up | 1,59E-08 |
| 836 | Homo sapiens caspase 3, apoptosis-related cysteine peptidase | 1,348 | up | 2,15E-09 |
| 900 | Homo sapiens cyclin G1 | 1,149 | up | 7,07E-16 |
| 8812 | Homo sapiens cyclin K | 1,155 | up | 1,47E-13 |
| 57018 | Homo sapiens cyclin L1 | 1,016 | up | 4,96E-07 |
| 4345 | Homo sapiens CD200 molecule | 1,395 | up | 1,74E-05 |
| 56990 | CDC42 small effector 2 | 1,376 | up | 9,68E-10 |
| 8558 | Homo sapiens cyclin-dependent kinase 10 | 1,068 | up | 2,75E-09 |
| 51755 | Homo sapiens cyclin-dependent kinase 12 | 1,086 | up | 2,90E-09 |
| 1018 | Homo sapiens cyclin-dependent kinase 3 | 1,252 | up | 5,90E-07 |
| 1060 | Homo sapiens centromere protein C | 1,078 | up | 8,59E-12 |
| 80152 | Homo sapiens centromere protein T | 1,837 | up | 9,69E-12 |
| 1195 | Homo sapiens CDC-like kinase 1 | 1,039 | up | 5,42E-11 |
| 51727 | Homo sapiens cytidine monophosphate (UMP-CMP) kinase 1, cytosolic | 1,037 | up | 1,27E-13 |
| 84766 | Homo sapiens calcium release activated channel regulator 2A | 1,831 | up | 3,24E-08 |
| 51379 | Homo sapiens cytokine receptor-like factor 3 | 1,040 | up | 5,11E-10 |
| 131544 | Homo sapiens beta-gamma crystallin domain containing 3 | 1,034 | up | 2,31E-09 |
| 1656 | Homo sapiens DEAD (Asp-Glu-Ala-Asp) box helicase 6 | 1,005 | up | 5,27E-11 |
| 163486 | Homo sapiens DENN/MADD domain containing 1B | 1,134 | up | 2,66E-10 |
| 10260 | Homo sapiens DENN/MADD domain containing 4A | 1,425 | up | 1,81E-10 |
| 54431 | Homo sapiens DnaJ (Hsp40) homolog, subfamily C, member 10 | 1,201 | up | 1,30E-17 |
| 144132 | Homo sapiens dynein heavy chain domain 1 | 1,214 | up | 2,91E-05 |
| 1788 | Homo sapiens DNA (cytosine-5-)-methyltransferase 3 alpha | 1,178 | up | 2,46E-13 |
| 150290 | Homo sapiens dual specificity phosphatase 18 | 1,370 | up | 3,20E-11 |
| 1876 | Homo sapiens E2F transcription factor 6 | 1,955 | up | 8,54E-10 |
| 26098 | Homo sapiens erythroid differentiation regulatory factor 1 | 1,395 | up | 1,80E-11 |
| 30008 | Homo sapiens EGF containing fibulin-like extracellular matrix protein 2 | 1,174 | up | 4,29E-07 |
| 79631 | Homo sapiens elongation factor Tu GTP binding domain containing 1 | 1,446 | up | 8,62E-12 |
| 2005 | Homo sapiens ELK4, ETS-domain protein (SRF accessory protein 1) | 1,142 | up | 4,52E-10 |
| 84173 | Homo sapiens ELMO/CED-12 domain containing 3 | 1,103 | up | 1,86E-08 |
| 146956 | Homo sapiens essential meiotic structure-specific endonuclease 1 | 1,115 | up | 1,15E-04 |
| 2053 | Homo sapiens epoxide hydrolase 2, cytoplasmic | 1,186 | up | 2,23E-08 |
| 79574 | Homo sapiens EPS8-like 3 (EPS8L3), transcript variant 1 | 1,296 | up | 5,07E-06 |
| 2130 | Homo sapiens EWS RNA-binding protein 1 | 1,323 | up | 7,41E-08 |
| 54932 | Homo sapiens exonuclease 3'-5' domain containing 3 | 1,622 | up | 5,45E-13 |
| 63901 | Homo sapiens family with sequence similarity 111, member A | 1,066 | up | 2,01E-11 |
| 159090 | Homo sapiens family with sequence similarity 122B | 1,438 | up | 7,26E-11 |
| 388581 | Homo sapiens family with sequence similarity 132, member A | 1,137 | up | 0,010997241 |
| 152756 | Homo sapiens family with sequence similarity 218, member A | 1,474 | up | 4,48E-13 |
| 253725 | Homo sapiens family with sequence similarity 21, member C | 1,144 | up | 1,03E-12 |
| 54537 | Homo sapiens family with sequence similarity 35, member A | 1,214 | up | 2,46E-12 |
| 11153 | Homo sapiens FIC domain containing | 1,096 | up | 3,98E-11 |
| 27086 | Homo sapiens forkhead box P1 | 1,070 | up | 9,54E-06 |
| 8939 | Homo sapiens far upstream element (FUSE) binding protein 3 | 1,520 | up | 5,63E-11 |
| 2550 | Homo sapiens gamma-aminobutyric acid (GABA) B receptor, 1 | 2,015 | up | 3,43E-09 |
| 2624 | Homo sapiens GATA binding protein 2 | 1,657 | up | 4,59E-05 |
| 23015 | Homo sapiens golgin A8 family, member A | 1,930 | up | 1,06E-08 |
| 122876 | Homo sapiens glycoprotein hormone beta 5 | 1,627 | up | 2,20E-05 |
| 84705 | Homo sapiens GTP binding protein 3 (mitochondrial) | 1,060 | up | 8,20E-10 |
| 3038 | Homo sapiens hyaluronan synthase 3 | 1,649 | up | 4,37E-10 |
| 29915 | Homo sapiens host cell factor C2 | 1,098 | up | 1,28E-09 |
| 283450 | Homo sapiens HECT domain containing E3 ubiquitin protein ligase 4 | 1,151 | up | 3,78E-08 |
| 8924 | Homo sapiens HECT and RLD domain containing E3 ubiquitin protein ligase 2 | 1,700 | up | 1,55E-10 |
| 284004 | Homo sapiens hexosaminidase (glycosyl hydrolase family 20, catalytic domain) containing | 1,197 | up | 4,09E-10 |
| 3007 | Homo sapiens histone cluster 1, H1d | 1,478 | up | 5,08E-13 |
| 3008 | Homo sapiens histone cluster 1, H1e | 1,285 | up | 1,44E-09 |
| 8338 | Homo sapiens histone cluster 2, H2ac | 1,412 | up | 2,30E-10 |
| 8349 | Homo sapiens histone cluster 2, H2be | 1,534 | up | 1,34E-07 |
| 90861 | Homo sapiens hematological and neurological expressed 1-like | 1,238 | up | 1,55E-11 |
| 144983 | Homo sapiens heterogeneous nuclear ribonucleoprotein A1-like 2 | 1,291 | up | 2,93E-12 |
| 84376 | Homo sapiens hook microtubule-tethering protein 3 | 1,001 | up | 1,07E-12 |
| 50809 | Homo sapiens heterochromatin protein 1, binding protein 3 | 1,177 | up | 2,79E-12 |
| 117245 | Homo sapiens HRAS-like suppressor family, member 5 | 1,144 | up | 4,41E-04 |
| 3292 | Homo sapiens hydroxysteroid (17-beta) dehydrogenase 1 | 1,633 | up | 6,02E-09 |
| 51144 | Homo sapiens hydroxysteroid (17-beta) dehydrogenase 12 | 1,046 | up | 6,09E-11 |
| 10075 | Homo sapiens HECT, UBA and WWE domain containing 1, E3 ubiquitin protein ligase | 1,193 | up | 2,77E-08 |
| 3483 | Homo sapiens insulin-like growth factor binding protein, acid labile subunit | 1,102 | up | 3,95E-09 |
| 8809 | Homo sapiens interleukin 18 receptor 1 | 1,032 | up | 5,28E-07 |
| 3559 | Homo sapiens interleukin 2 receptor, alpha | 1,161 | up | 6,91E-08 |
| 3566 | Homo sapiens interleukin 4 receptor | 1,517 | up | 1,10E-04 |
| 84289 | Homo sapiens inhibitor of growth family, member 5 | 1,003 | up | 1,56E-09 |
| 27130 | Homo sapiens inversin | 1,325 | up | 2,15E-08 |
| 3671 | Homo sapiens immunoglobulin superfamily containing leucine-rich repeat | 2,152 | up | 1,21E-06 |
| 3676 | Homo sapiens integrin, alpha 4 (antigen CD49D, alpha 4 subunit of VLA-4 receptor) | 1,180 | up | 1,45E-14 |
| 3700 | Homo sapiens inter-alpha-trypsin inhibitor heavy chain family, member 4 | 1,724 | up | 2,47E-08 |
| 8514 | Homo sapiens potassium channel, voltage gated subfamily A regulatory beta subunit 2 | 1,172 | up | 1,27E-08 |
| 56660 | Homo sapiens potassium channel, two pore domain subfamily K, member 12 | 1,229 | up | 2,82E-07 |
| 80853 | Homo sapiens lysine (K)-specific demethylase 7A | 1,098 | up | 5,42E-08 |
| 8570 | Homo sapiens KH-type splicing regulatory protein | 1,483 | up | 2,16E-10 |
| 4297 | Homo sapiens lysine (K)-specific methyltransferase 2A | 1,048 | up | 1,70E-08 |
| 474170 | Homo sapiens leucine rich repeat containing 37, member A2 | 1,374 | up | 4,35E-10 |
| 84230 | Homo sapiens leucine rich repeat containing 8 family, member C | 1,014 | up | 5,11E-08 |
| 23499 | Homo sapiens microtubule-actin crosslinking factor 1 | 1,053 | up | 3,47E-08 |
| 4134 | Homo sapiens microtubule-associated protein 4 | 1,626 | up | 2,69E-08 |
| 4154 | Homo sapiens muscleblind-like splicing regulator 1 | 1,001 | up | 9,77E-13 |
| 254394 | Homo sapiens minichromosome maintenance complex component 9 | 1,238 | up | 9,98E-10 |
| 56890 | Homo sapiens Mdm1 nuclear protein homolog (mouse) | 1,053 | up | 2,41E-12 |
| 4194 | Homo sapiens MDM4, p53 regulator | 1,004 | up | 8,64E-09 |
| 27304 | Homo sapiens molybdenum cofactor synthesis 3 | 1,392 | up | 4,02E-07 |
| 64432 | Homo sapiens mitochondrial ribosomal protein S25 | 1,370 | up | 1,57E-09 |
| 4485 | Homo sapiens macrophage stimulating 1 (hepatocyte growth factor-like) | 1,535 | up | 2,98E-09 |
| 100463488 | Homo sapiens MT-RNR2-like 10 | 1,581 | up | 2,77E-04 |
| 53904 | Homo sapiens myosin IIIA | 3,021 | up | 6,15E-09 |
| 90634 | Homo sapiens NEDD4 binding protein 2-like 1 | 1,030 | up | 1,32E-11 |
| 79612 | Homo sapiens N(alpha)-acetyltransferase 16, NatA auxiliary subunit | 1,112 | up | 3,03E-11 |
| 133686 | Homo sapiens NAD kinase 2, mitochondrial | 1,822 | up | 1,31E-12 |
| 100132406 | Homo sapiens neuroblastoma breakpoint family, member 10 | 1,004 | up | 1,41E-09 |
| 728841 | Homo sapiens neuroblastoma breakpoint family, member 8 | 1,040 | up | 7,22E-10 |
| 400818 | Homo sapiens mRNA; cDNA DKFZp686H059 (from clone DKFZp686H059) | 1,163 | up | 1,67E-11 |
| 79661 | Homo sapiens nei endonuclease VIII-like 1 (E. coli) | 1,332 | up | 1,97E-07 |
| 4820 | Homo sapiens natural killer cell triggering receptor | 1,145 | up | 5,71E-11 |
| 55035 | Homo sapiens nucleolar protein 8 | 1,235 | up | 3,37E-14 |
| 10811 | Homo sapiens NADPH oxidase activator 1 | 1,112 | up | 2,58E-08 |
| 27031 | Homo sapiens nephronophthisis 3 (adolescent) | 1,569 | up | 4,32E-08 |
| 4905 | Homo sapiens N-ethylmaleimide-sensitive factor | 1,205 | up | 1,65E-09 |
| 55916 | Homo sapiens nuclear transport factor 2-like export factor 2 | 1,025 | up | 1,21E-10 |
| 114883 | oxysterol binding protein-like 9 | 1,228 | up | 1,31E-11 |
| 64175 | Homo sapiens prolyl 3-hydroxylase 1 | 1,545 | up | 2,19E-10 |
| 80336 | Homo sapiens poly(A) binding protein, cytoplasmic 1-like | 1,919 | up | 1,82E-09 |
| 5083 | Homo sapiens paired box 9 | 2,639 | up | 5,28E-12 |
| 94104 | Homo sapiens PAX3 and PAX7 binding protein 1 | 1,224 | up | 4,71E-12 |
| 57526 | Homo sapiens protocadherin 19 | 1,930 | up | 2,85E-06 |
| 9659 | Homo sapiens phosphodiesterase 4D interacting protein | 1,856 | up | 1,99E-11 |
| 5150 | Homo sapiens phosphodiesterase 7A | 1,268 | up | 9,35E-13 |
| 23133 | Homo sapiens PHD finger protein 8 | 1,137 | up | 6,85E-10 |
| 5290 | Homo sapiens phosphatidylinositol-4,5-bisphosphate 3-kinase, catalytic subunit alpha | 1,156 | up | 4,27E-13 |
| 5310 | Homo sapiens polycystic kidney disease 1 (autosomal dominant) | 1,874 | up | 3,24E-10 |
| 5586 | Homo sapiens protein kinase N2 | 1,291 | up | 3,57E-09 |
| 5339 | Homo sapiens plectin | 1,918 | up | 7,46E-11 |
| 152926 | protein phosphatase, Mg2+/Mn2+ dependent, 1K | 1,968 | up | 2,29E-11 |
| 51400 | Homo sapiens protein phosphatase methylesterase 1 | 1,491 | up | 3,96E-11 |
| 5567 | Homo sapiens protein kinase, cAMP-dependent, catalytic, beta | 1,725 | up | 6,74E-07 |
| 8899 | Homo sapiens pre-mRNA processing factor 4B | 1,337 | up | 1,70E-11 |
| 5711 | Homo sapiens proteasome (prosome, macropain) 26S subunit, non-ATPase, 5 | 1,434 | up | 3,56E-12 |
| 9444 | Homo sapiens QKI, KH domain containing, RNA binding | 1,137 | up | 4,44E-11 |
| 23637 | Homo sapiens RAB GTPase activating protein 1 | 1,117 | up | 1,99E-10 |
| 11159 | Homo sapiens RAB, member of RAS oncogene family-like 2A | 1,155 | up | 7,24E-11 |
| 10743 | Homo sapiens retinoic acid induced 1 | 1,300 | up | 2,47E-05 |
| 9693 | Homo sapiens Rap guanine nucleotide exchange factor (GEF) 2 | 1,017 | up | 1,62E-04 |
| 51735 | Homo sapiens Rap guanine nucleotide exchange factor (GEF) 6 | 1,176 | up | 2,28E-12 |
| 5924 | Homo sapiens Ras protein-specific guanine nucleotide-releasing factor 2 | 1,415 | up | 1,76E-08 |
| 11123 | Homo sapiens RCAN family member 3 | 1,205 | up | 6,79E-09 |
| 1104 | Homo sapiens regulator of chromosome condensation 1 | 1,088 | up | 2,47E-08 |
| 5978 | Homo sapiens RE1-silencing transcription factor | 1,050 | up | 2,03E-12 |
| 6248 | Homo sapiens regulatory solute carrier protein, family 1, member 1 | 1,193 | up | 2,83E-13 |
| 146760 | Homo sapiens reticulon 4 receptor-like 1 | 1,596 | up | 1,23E-04 |
| 6284 | Homo sapiens S100 calcium binding protein A13 | 1,229 | up | 5,40E-08 |
| 9672 | Homo sapiens syndecan 3 | 2,110 | up | 2,16E-09 |
| 57337 | Homo sapiens SUMO1/sentrin specific peptidase 7 | 1,069 | up | 1,51E-08 |
| 10801 | Homo sapiens septin 9 | 1,748 | up | 5,04E-16 |
| 6497 | Homo sapiens v-ski avian sarcoma viral oncogene homolog | 1,311 | up | 5,79E-14 |
| 91137 | Homo sapiens solute carrier family 25, member 46 | 1,089 | up | 1,57E-11 |
| 6599 | Homo sapiens SWI/SNF related, matrix associated, actin dependent regulator of chromatin, subfamily c, member 1 | 1,044 | up | 4,91E-12 |
| 23049 | Homo sapiens SMG1 phosphatidylinositol 3-kinase-related kinase | 1,536 | up | 1,38E-14 |
| 23381 | Homo sapiens SMG5 nonsense mediated mRNA decay factor | 1,207 | up | 1,23E-09 |
| 6651 | Homo sapiens SON DNA binding protein | 1,413 | up | 3,43E-15 |
| 23013 | Homo sapiens spen family transcriptional repressor | 1,342 | up | 6,78E-10 |
| 647135 | Homo sapiens SLIT-ROBO Rho GTPase activating protein 2B | 1,169 | up | 6,38E-14 |
| 23524 | Homo sapiens serine/arginine repetitive matrix 2 | 1,707 | up | 2,29E-14 |
| 9295 | Homo sapiens serine/arginine-rich splicing factor 11 | 1,169 | up | 2,86E-11 |
| 8869 | Homo sapiens ST3 beta-galactoside alpha-2,3-sialyltransferase 5 | 1,309 | up | 2,04E-14 |
| 8027 | Homo sapiens signal transducing adaptor molecule (SH3 domain and ITAM motif) 1 | 1,626 | up | 5,06E-09 |
| 23353 | Homo sapiens Sad1 and UNC84 domain containing 1 | 1,189 | up | 8,65E-09 |
| 23224 | Homo sapiens spectrin repeat containing, nuclear envelope 2 | 1,241 | up | 2,90E-09 |
| 259293 | Homo sapiens taste receptor, type 2, member 30 | 1,295 | up | 1,55E-05 |
| 26000 | Homo sapiens TBC1 domain family, member 10B | 1,223 | up | 2,44E-10 |
| 6917 | Homo sapiens transcription elongation factor A (SII), 1 | 1,226 | up | 2,00E-14 |
| 9967 | Homo sapiens thyroid hormone receptor associated protein 3 | 1,061 | up | 2,11E-09 |
| 11011 | Homo sapiens tousled-like kinase 2 | 1,033 | up | 2,78E-11 |
| 283578 | Homo sapiens transmembrane emp24 protein transport domain containing 8 | 1,680 | up | 1,28E-09 |
| 645369 | transmembrane protein 200C | 1,575 | up | 1,64E-04 |
| 339456 | Homo sapiens transmembrane protein 52 | 1,056 | up | 1,60E-07 |
| 10188 | Homo sapiens tyrosine kinase, non-receptor, 2 | 1,728 | up | 1,13E-09 |
| 7140 | Homo sapiens troponin T type 3 (skeletal, fast) | 1,509 | up | 2,58E-05 |
| 90313 | Homo sapiens tumor protein p53 inducible protein 13 | 1,052 | up | 3,31E-09 |
| 7169 | Homo sapiens tropomyosin 2 (beta) | 1,748 | up | 1,08E-07 |
| 22906 | trafficking protein, kinesin binding 1 | 1,248 | up | 1,47E-05 |
| 51095 | Homo sapiens tRNA nucleotidyl transferase, CCA-adding, 1 | 1,023 | up | 3,27E-08 |
| 7249 | Homo sapiens tuberous sclerosis 2 | 1,615 | up | 8,57E-15 |
| 7273 | Homo sapiens titin | 1,951 | up | 2,12E-10 |
| 9100 | Homo sapiens ubiquitin specific peptidase 10 | 1,382 | up | 4,05E-09 |
| 7402 | Homo sapiens utrophin | 1,579 | up | 4,98E-16 |
| 57654 | UV-stimulated scaffold protein A | 1,294 | up | 8,07E-08 |
| 7422 | Homo sapiens vascular endothelial growth factor A | 1,527 | up | 5,80E-10 |
| 389668 | Homo sapiens XK, Kell blood group complex subunit-related family, member 9 | 1,396 | up | 4,85E-10 |
| 8531 | Homo sapiens Y box binding protein 3 | 1,063 | up | 4,85E-11 |
| 27033 | Homo sapiens zinc finger and BTB domain containing 32 | 1,688 | up | 7,53E-06 |
| 9877 | Homo sapiens zinc finger CCCH-type containing 11A | 1,007 | up | 2,72E-09 |
| 23318 | zinc finger, CCHC domain containing 11 | 1,110 | up | 3,60E-05 |
| 9839 | Homo sapiens zinc finger E-box binding homeobox 2 | 1,358 | up | 9,43E-15 |
| 55345 | Homo sapiens zinc finger, GRF-type containing 1 | 1,550 | up | 2,82E-11 |
| 284307 | Homo sapiens zinc finger protein interacting with K protein 1 | 1,113 | up | 2,24E-10 |
| 84460 | Homo sapiens zinc finger, matrin-type 1 | 1,878 | up | 8,79E-07 |
| 10771 | Homo sapiens zinc finger, MYND-type containing 11 | 1,562 | up | 2,38E-13 |
| 7700 | Homo sapiens zinc finger protein 141 | 1,097 | up | 6,92E-12 |
| 90338 | Homo sapiens zinc finger protein 160 | 1,304 | up | 2,07E-14 |
| 7587 | Homo sapiens zinc finger protein 37A | 1,244 | up | 3,67E-10 |
| 79744 | Homo sapiens zinc finger protein 419 | 1,346 | up | 1,04E-10 |
| 147657 | Homo sapiens zinc finger protein 480 | 1,126 | up | 1,26E-11 |
| 162972 | Homo sapiens zinc finger protein 550 | 1,793 | up | 2,22E-10 |
| 148156 | zinc finger protein 558 | 1,092 | up | 1,12E-08 |
| 284370 | Homo sapiens zinc finger protein 615 | 1,229 | up | 4,99E-13 |
| 55657 | Homo sapiens zinc finger protein 692 | 1,062 | up | 1,60E-08 |
| 347344 | Homo sapiens zinc finger protein 81 | 1,690 | up | 1,40E-08 |
| 53947 | alpha 1,4-galactosyltransferase | -1,819 | down | 4,78E-13 |
| 55347 | Homo sapiens abhydrolase domain containing 10 | -1,143 | down | 0,009798489 |
| 345651 | Homo sapiens actin, beta-like 2 | -1,137 | down | 2,52E-08 |
| 71 | Homo sapiens actin gamma 1 | -1,217 | down | 6,48E-11 |
| 1645 | aldo-keto reductase family 1, member C1 | -1,477 | down | 7,29E-07 |
| 29123 | Homo sapiens ankyrin repeat domain 11 | -2,283 | down | 9,44E-15 |
| 23253 | Homo sapiens ankyrin repeat domain 12 | -2,123 | down | 2,08E-08 |
| 8125 | Homo sapiens acidic (leucine-rich) nuclear phosphoprotein 32 family, member A | -2,413 | down | 1,83E-11 |
| 23519 | Homo sapiens acidic (leucine-rich) nuclear phosphoprotein 32 family, member D | -1,275 | down | 2,72E-09 |
| 11154 | Homo sapiens adaptor-related protein complex 4, sigma 1 subunit | -1,693 | down | 6,50E-06 |
| 80832 | Homo sapiens apolipoprotein L, 4 | -1,902 | down | 3,16E-08 |
| 480 | Homo sapiens ATPase, Na+/K+ transporting, alpha 4 polypeptide | -1,365 | down | 1,34E-08 |
| 267020 | Homo sapiens ATP synthase, H+ transporting, mitochondrial Fo complex, subunit G2 | -2,409 | down | 1,47E-21 |
| 9530 | Homo sapiens BCL2-associated athanogene 4 | -1,622 | down | 2,91E-05 |
| 9275 | Homo sapiens B-cell CLL/lymphoma 7B | -1,799 | down | 2,48E-16 |
| 128408 | Homo sapiens basic helix-loop-helix family, member e23 | -1,709 | down | 2,70E-09 |
| 11120 | Homo sapiens butyrophilin, subfamily 2, member A1 | -1,664 | down | 2,86E-08 |
| 810 | Homo sapiens calmodulin-like 3 | -1,016 | down | 5,75E-06 |
| 838 | Homo sapiens caspase 5, apoptosis-related cysteine peptidase | -1,503 | down | 2,47E-10 |
| 885 | Homo sapiens cholecystokinin | -1,614 | down | 0,001755532 |
| 11314 | Homo sapiens CD300a molecule | -2,289 | down | 2,52E-15 |
| 148170 | Homo sapiens CDC42 effector protein (Rho GTPase binding) 5 | -1,303 | down | 8,70E-09 |
| 728642 | Homo sapiens cyclin-dependent kinase 11A | -1,393 | down | 2,39E-10 |
| 64105 | Homo sapiens centromere protein K | -1,888 | down | 2,17E-08 |
| 146845 | Homo sapiens cilia and flagella associated protein 52 | -2,011 | down | 2,29E-12 |
| 1154 | Homo sapiens cytokine inducible SH2-containing protein | -1,465 | down | 2,03E-09 |
| 1198 | Homo sapiens CDC-like kinase 3 | -2,246 | down | 2,65E-18 |
| 9377 | Homo sapiens cytochrome c oxidase subunit Va | -2,376 | down | 1,15E-19 |
| 1399 | Homo sapiens v-crk avian sarcoma virus CT10 oncogene homolog-like | -2,011 | down | 6,03E-11 |
| 1407 | Homo sapiens cryptochrome circadian clock 1 | -1,194 | down | 1,30E-05 |
| 1520 | Homo sapiens cathepsin S | -2,813 | down | 3,37E-16 |
| 54205 | Homo sapiens cytochrome c, somatic | -1,457 | down | 4,33E-12 |
| 267012 | Homo sapiens D-amino acid oxidase activator | -1,672 | down | 2,45E-09 |
| 1633 | Homo sapiens deoxycytidine kinase | -2,066 | down | 2,57E-13 |
| 55789 | Homo sapiens DEP domain containing 1B | -1,492 | down | 5,46E-08 |
| 3338 | Homo sapiens DnaJ (Hsp40) homolog, subfamily C, member 4 | -1,425 | down | 5,43E-06 |
| 1842 | extracellular matrix protein 2, female organ and adipocyte specific | -1,098 | down | 0,00185457 |
| 128178 | Homo sapiens EDAR-associated death domain | -1,733 | down | 3,35E-10 |
| 1915 | Homo sapiens eukaryotic translation elongation factor 1 alpha 1 | -1,329 | down | 2,10E-10 |
| 2115 | Homo sapiens ets variant 1 (ETV1), transcript variant 1 | -1,336 | down | 1,11E-07 |
| 51439 | Homo sapiens family with sequence similarity 8, member A1 | -2,935 | down | 3,27E-15 |
| 51077 | Homo sapiens FCF1 rRNA-processing protein | -1,241 | down | 1,93E-10 |
| 10875 | Homo sapiens fibrinogen-like 2 | -2,161 | down | 1,32E-19 |
| 116113 | Homo sapiens forkhead box P4 | -1,065 | down | 7,47E-08 |
| 53940 | Homo sapiens ferritin, heavy polypeptide-like 17 | -2,834 | down | 1,96E-16 |
| 2591 | Homo sapiens polypeptide N-acetylgalactosaminyltransferase 3 | -1,778 | down | 7,75E-12 |
| 8484 | Homo sapiens galanin receptor 3 | -1,546 | down | 2,28E-08 |
| 92292 | Homo sapiens glycine-N-acyltransferase-like 1 | -1,342 | down | 0,017005743 |
| 285601 | Homo sapiens G protein-coupled receptor 150 | -1,120 | down | 2,93E-07 |
| 2914 | Homo sapiens glutamate receptor, metabotropic 4 | -1,890 | down | 2,41E-06 |
| 610 | Homo sapiens hyperpolarization activated cyclic nucleotide gated potassium channel 2 | -1,984 | down | 8,74E-12 |
| 25994 | Homo sapiens HIG1 hypoxia inducible domain family, member 1A | -2,170 | down | 2,84E-15 |
| 28996 | Homo sapiens homeodomain interacting protein kinase 2 | -1,209 | down | 1,12E-05 |
| 8350 | Homo sapiens histone cluster 1, H3a | -2,115 | down | 2,25E-14 |
| 8353 | Homo sapiens histone cluster 1, H3e | -1,705 | down | 1,42E-12 |
| 8356 | Homo sapiens histone cluster 1, H3j | -2,087 | down | 2,73E-11 |
| 653604 | Homo sapiens histone cluster 2, H3d | -1,618 | down | 6,83E-12 |
| 220988 | Homo sapiens heterogeneous nuclear ribonucleoprotein A3 | -2,873 | down | 7,71E-14 |
| 4670 | Homo sapiens heterogeneous nuclear ribonucleoprotein M | -1,841 | down | 5,92E-18 |
| 3223 | Homo sapiens homeobox C6 | -1,149 | down | 0,035598706 |
| 11255 | Homo sapiens histamine receptor H3 | -1,540 | down | 7,91E-04 |
| 3336 | heat shock 10kDa protein 1 | -1,300 | down | 6,15E-14 |
| 9445 | Homo sapiens integral membrane protein 2B | -2,708 | down | 1,56E-17 |
| 83700 | Homo sapiens junctional adhesion molecule 3 | -1,456 | down | 3,22E-06 |
| 3785 | Homo sapiens potassium channel, voltage gated KQT-like subfamily Q, member 2 | -1,857 | down | 1,96E-08 |
| 80856 | Homo sapiens KIAA1715 | -1,164 | down | 0,003565264 |
| 54741 | Homo sapiens leptin receptor overlapping transcript | -1,079 | down | 2,93E-06 |
| 4193 | Homo sapiens MDM2 proto-oncogene, E3 ubiquitin protein ligase | -1,445 | down | 3,57E-08 |
| 143098 | Homo sapiens membrane protein, palmitoylated 7 | -1,303 | down | 4,52E-10 |
| 100288485 | Homo sapiens MT-RNR2-like 7 | -1,276 | down | 0,008498585 |
| 10398 | Homo sapiens myosin, light chain 9, regulatory | -1,467 | down | 0,008767899 |
| 1482 | Homo sapiens NK2 homeobox 5 (NKX2-5), transcript variant 2 | -1,188 | down | 2,15E-09 |
| 64802 | Homo sapiens nicotinamide nucleotide adenylyltransferase 1 | -1,194 | down | 9,86E-10 |
| 256933 | Homo sapiens neuropeptide B | -1,126 | down | 6,91E-10 |
| 261734 | Homo sapiens nephronophthisis 4 | -1,547 | down | 2,38E-08 |
| 140767 | Homo sapiens neurensin 1 | -1,003 | down | 0,006942097 |
| 5036 | Homo sapiens proliferation-associated 2G4, 38kDa | -1,854 | down | 7,64E-09 |
| 5042 | Homo sapiens poly(A) binding protein, cytoplasmic 3 | -2,430 | down | 8,02E-14 |
| 390928 | Homo sapiens iron/zinc purple acid phosphatase-like protein | -1,575 | down | 6,95E-10 |
| 55795 | Homo sapiens PCI domain containing 2 | -1,553 | down | 1,88E-12 |
| 27344 | Homo sapiens proprotein convertase subtilisin/kexin type 1 inhibitor | -1,301 | down | 1,20E-05 |
| 5194 | Homo sapiens peroxisomal biogenesis factor 13 | -1,567 | down | 2,54E-10 |
| 441531 | Homo sapiens phosphoglycerate mutase family member 4 | -1,904 | down | 2,70E-17 |
| 9749 | Homo sapiens phosphatase and actin regulator 2 | -2,039 | down | 3,83E-12 |
| 55041 | Homo sapiens pleckstrin homology domain containing, family B (evectins) member 2 | -1,531 | down | 1,48E-15 |
| 87178 | Homo sapiens polyribonucleotide nucleotidyltransferase 1 | -2,047 | down | 9,23E-09 |
| 5423 | Homo sapiens polymerase (DNA directed), beta | -1,015 | down | 3,10E-07 |
| 5478 | Homo sapiens peptidylprolyl isomerase A (cyclophilin A) | -1,679 | down | 2,80E-15 |
| 653505 | Homo sapiens peptidylprolyl isomerase A (cyclophilin A)-like 4B | -1,346 | down | 2,41E-11 |
| 644591 | Homo sapiens peptidylprolyl isomerase A (cyclophilin A)-like 4G | -2,688 | down | 6,78E-17 |
| 5504 | Homo sapiens protein phosphatase 1, regulatory (inhibitor) subunit 2 | -2,495 | down | 3,93E-17 |
| 9701 | Homo sapiens protein phosphatase 6, regulatory subunit 2 | -2,022 | down | 2,40E-11 |
| 221823 | Homo sapiens phosphoribosyl pyrophosphate synthetase 1-like 1 | -2,362 | down | 2,76E-14 |
| 54458 | Homo sapiens proline rich 13 | -1,391 | down | 7,95E-10 |
| 80164 | Homo sapiens proline rich 36 | -1,818 | down | 3,22E-14 |
| 5700 | Homo sapiens proteasome (prosome, macropain) 26S subunit, ATPase, 1 | -1,210 | down | 8,84E-11 |
| 5725 | Homo sapiens polypyrimidine tract binding protein 1 | -2,498 | down | 1,57E-13 |
| 10728 | Homo sapiens prostaglandin E synthase 3 (cytosolic) | -2,075 | down | 1,66E-16 |
| 5781 | Homo sapiens protein tyrosine phosphatase, non-receptor type 11 | -2,188 | down | 2,32E-14 |
| 10411 | Rap guanine nucleotide exchange factor (GEF) 3 | -1,332 | down | 6,53E-08 |
| 91179 | Homo sapiens scavenger receptor class F, member 2 | -1,277 | down | 1,04E-08 |
| 9805 | Homo sapiens secernin 1 | -1,333 | down | 2,45E-07 |
| 6418 | Homo sapiens SET nuclear proto-oncogene | -1,824 | down | 4,39E-05 |
| 84193 | Homo sapiens SET domain containing 3 | -1,081 | down | 1,69E-09 |
| 6493 | Homo sapiens single-minded family bHLH transcription factor 2 | -1,963 | down | 2,80E-15 |
| 63910 | Homo sapiens solute carrier family 17 (vesicular nucleotide transporter), member 9 | -1,770 | down | 4,56E-06 |
| 6635 | Homo sapiens small nuclear ribonucleoprotein polypeptide E | -1,853 | down | 9,76E-19 |
| 23514 | scaffolding protein involved in DNA repair | -1,311 | down | 0,002361269 |
| 139886 | Homo sapiens spindlin family, member 4 | -1,603 | down | 7,28E-10 |
| 144108 | Homo sapiens SPT2, Suppressor of Ty, domain containing 1 (S. cerevisiae) | -1,322 | down | 8,63E-14 |
| 6725 | Homo sapiens src-related kinase lacking C-terminal regulatory tyrosine and N-terminal myristylation sites | -2,540 | down | 2,20E-13 |
| 6726 | Homo sapiens signal recognition particle 9kDa | -3,485 | down | 6,79E-18 |
| 548313 | Homo sapiens synovial sarcoma, X breakpoint 4B | -1,877 | down | 3,64E-10 |
| 6613 | Homo sapiens small ubiquitin-like modifier 2 | -2,200 | down | 5,42E-22 |
| 6836 | Homo sapiens surfeit 4 (SURF4), transcript variant 1 | -1,172 | down | 1,12E-13 |
| 258010 | Homo sapiens small VCP/p97-interacting protein | -1,776 | down | 4,24E-07 |
| 6950 | Homo sapiens t-complex 1 | -2,272 | down | 3,88E-09 |
| 7013 | Homo sapiens telomeric repeat binding factor (NIMA-interacting) 1 | -1,207 | down | 1,83E-09 |
| 7035 | tissue factor pathway inhibitor (lipoprotein-associated coagulation inhibitor) | -1,691 | down | 1,61E-06 |
| 7039 | Homo sapiens transforming growth factor, alpha | -1,166 | down | 4,39E-06 |
| 53346 | Homo sapiens transmembrane 6 superfamily member 1 | -2,395 | down | 2,40E-11 |
| 79073 | Homo sapiens transmembrane protein 109 | -1,514 | down | 2,98E-13 |
| 7178 | Homo sapiens tumor protein, translationally-controlled 1 | -1,963 | down | 1,71E-18 |
| 7187 | Homo sapiens TNF receptor-associated factor 3 | -2,271 | down | 2,39E-13 |
| 84231 | Homo sapiens TNF receptor-associated factor 7, E3 ubiquitin protein ligase | -3,011 | down | 8,19E-18 |
| 55809 | Homo sapiens transcriptional regulating factor 1 | -2,647 | down | 8,62E-16 |
| 9830 | Homo sapiens tripartite motif containing 14 | -1,567 | down | 2,86E-12 |
| 129868 | Homo sapiens tripartite motif containing 43 | -1,864 | down | 5,62E-10 |
| 7225 | Homo sapiens transient receptor potential cation channel, subfamily C, member 6 | -1,164 | down | 4,23E-08 |
| 7278 | Homo sapiens tubulin, alpha 3c | -1,405 | down | 5,03E-07 |
| 10054 | Homo sapiens ubiquitin-like modifier activating enzyme 2 | -2,027 | down | 1,47E-20 |
| 9898 | Homo sapiens ubiquitin associated protein 2-like | -1,274 | down | 7,34E-11 |
| 130507 | Homo sapiens ubiquitin protein ligase E3 component n-recognin 3 (putative) | -2,160 | down | 6,95E-13 |
| 2837 | Homo sapiens urotensin 2 receptor | -1,685 | down | 2,87E-08 |
| 10810 | Homo sapiens WAS protein family, member 3 | -1,295 | down | 5,97E-05 |
| 51384 | Homo sapiens wingless-type MMTV integration site family, member 16 | -1,775 | down | 2,17E-08 |
| 9589 | Homo sapiens Wilms tumor 1 associated protein | -1,084 | down | 2,39E-07 |
| 79670 | zinc finger, CCHC domain containing 6 | -1,467 | down | 3,56E-07 |
| 85437 | Homo sapiens zinc finger CCHC-type and RNA binding motif 1 | -2,322 | down | 7,89E-14 |
| 64393 | Homo sapiens zinc finger, matrin-type 3 | -1,266 | down | 3,31E-14 |
| 57567 | Homo sapiens zinc finger protein 319 | -2,751 | down | 1,91E-16 |
| 163051 | Homo sapiens zinc finger protein 709 | -1,066 | down | 1,80E-08 |
| 283337 | Homo sapiens zinc finger protein 740 | -1,422 | down | 8,23E-12 |
