# Supplementary material for: Inflammation and regulatory T cell genes are differentially expressed in peripheral blood mononuclear cells of Parkinson’s disease patients
Source: Sci Rep. 2021 Jan 27;11:2316. doi: 10.1038/s41598-021-81961-7 (PMC7841172; doi:10.1038/s41598-021-81961-7)
Supplement: Supplementary file 2 — Supplementary Table 1. [file 41598_2021_81961_MOESM2_ESM.docx]

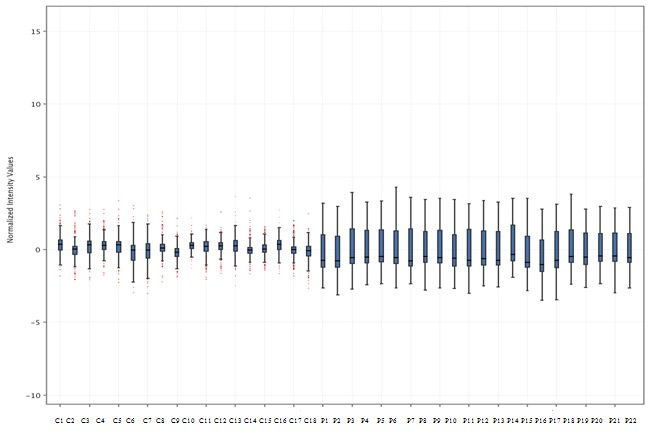


**Supplementary Figure 1:** Boxplots for normalized gene expression data (*P indicates PD patients, C indicates controls).*


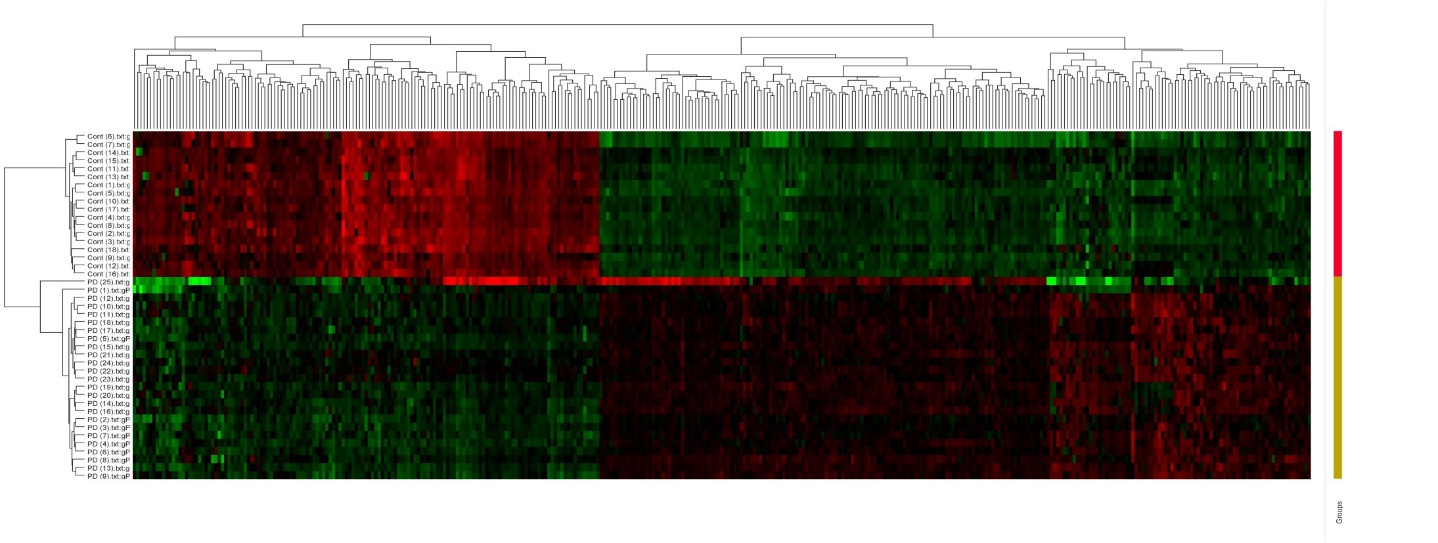


**Supplementary Figure 2:** Hierarchical clustering heatmap between PD patients and controls. Red indicates that relatively upregulated genes, green indicates relatively downregulated genes and black indicates the genes that show no significant changes in expression.


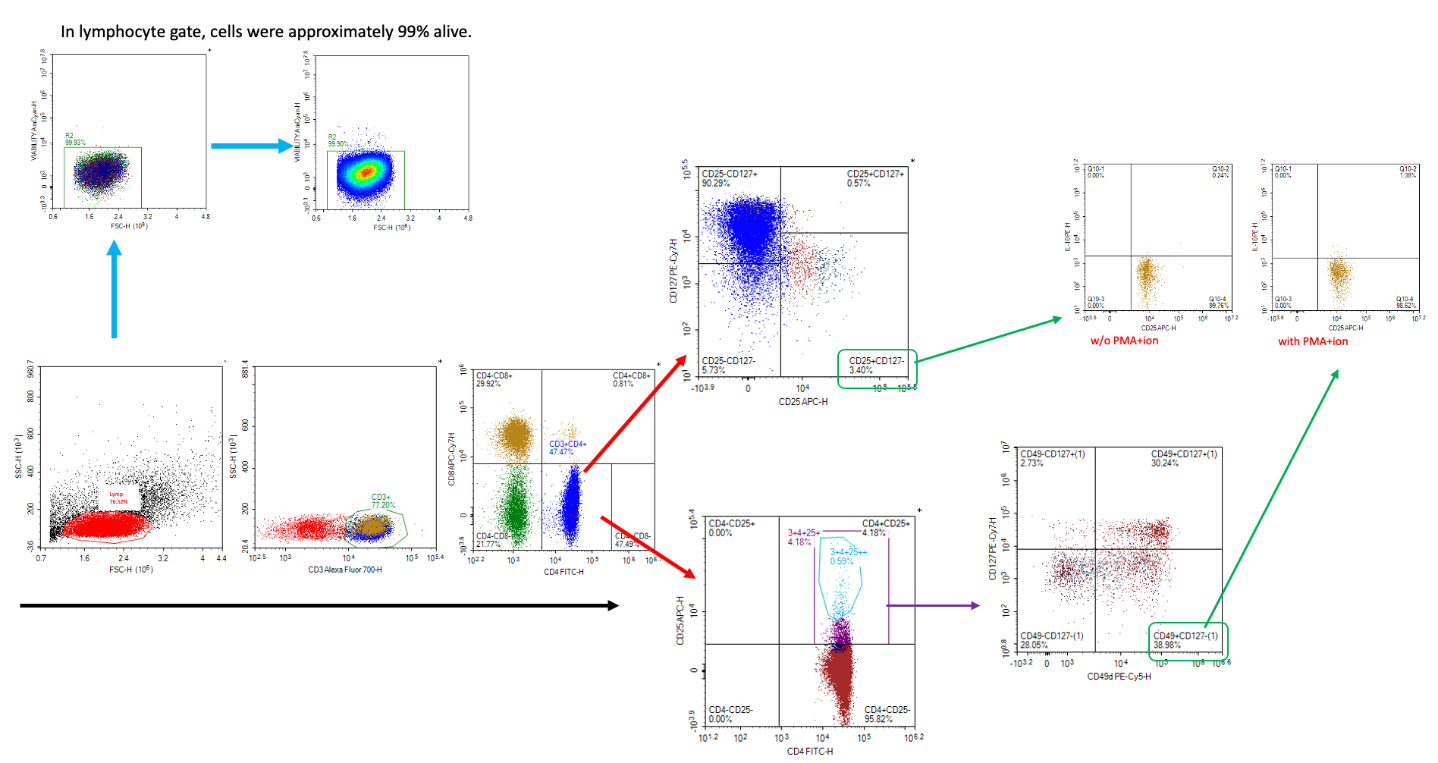


**Supplementary Figure 3: Displaying of the gating strategy**
